# Rubredoxin 1 Is Required for Formation of the Functional Photosystem II Core Complex in *Arabidopsis thaliana*

**DOI:** 10.3389/fpls.2022.824358

**Published:** 2022-02-23

**Authors:** Liping Che, Han Meng, Junxiang Ruan, Lianwei Peng, Lin Zhang

**Affiliations:** ^1^School of Environmental and Geographical Sciences, Shanghai Normal University, Shanghai, China; ^2^Shanghai Key Laboratory of Plant Molecular Sciences, College of Life Sciences, Shanghai Normal University, Shanghai, China

**Keywords:** rubredoxin, photosystem II, reaction center, *psbA*, ribosome profiling, *Arabidopsis thaliana*

## Abstract

Chloroplast thylakoid protein rubredoxin 1 (RBD1) in Chlamydomonas and its cyanobacterial homolog RubA contain a rubredoxin domain. These proteins have been proposed to participate in the assembly of photosystem II (PSII) at early stages. However, the effects of inactivation of RBD1 on PSII assembly in higher plants are largely unclear. Here, we characterized an Arabidopsis *rbd1* mutant in detail. A drastic reduction of intact PSII complex but relatively higher levels of assembly intermediates including PSII RC, pre-CP47, and pre-CP43 were found in *rbd1*. Polysome association and ribosome profiling revealed that ribosome recruitment of *psbA* mRNA is specifically reduced. Consistently, *in vivo* protein pulse-chase labeling showed that the rate of D1/pD1 synthesis is significantly reduced in *rbd1* compared with WT. Moreover, newly synthesized mature D1 and pD1/D2 can assemble into the PSII reaction center (RC) complex but further formation of larger PSII complexes is nearly totally blocked in *rbd1*. Our data imply that RBD1 is not only required for the formation of a functional PSII core complex during the early stages of PSII assembly but may also be involved in the translation of D1 in higher plants.

## Introduction

Photosystem II (PSII) is the primary pigment-protein complex in the photosynthetic electron transport chain in oxygenic organisms. PSII harvests light energy to split water resulting in the release of oxygen and electrons that are transferred from water to plastoquinone in cyanobacteria, algae, and plants. PSII drives oxygenic photosynthesis together with PSI (Photosystem I), Cyt *b_6_f* (Cytochrome *b_6_f*), and ATP synthase, and provides energy and oxygen for most living organisms on earth (Nelson and Junge, 2015; Shen, 2015). In higher plants, PSII is composed of the PSII reaction center (RC), CP43 and CP47, the oxygen-evolving complex (OEC), and LHCII as well as several small subunits with a low molecular mass. PSII RC is the minimum unit for the primary photochemical reaction and is composed of the subunits D1, D2, PsbI, and two subunits of cytochrome *b*_559_ (PsbE and PsbF) (Nelson and Yocum, 2006). The D1 and D2 proteins form a heterodimer and bind the basal redox components required for PSII electron transport such as P680, pheophytin, non-heme iron and quinone (Umena et al., 2011). CP43 and CP47 are peripheral core antenna subunits attached to the PSII RC. They function in energy transfer from light harvesting complex (LHCII) to the PSII reaction center. As the catalytic site for water splitting, OEC is composed of one Ca atom, four Mn atoms, and five oxygen atoms (Mn_4_CaO_5_ cluster). Seven amino acid residues from D1 and CP43 provide ligands to OEC (Umena et al., 2011; Shen, 2015). PsbO, PsbP, and PsbQ are attached to the lumen side of PSII. Together with CP43, these proteins provide protection and contribute to access channels for substrate and products during water oxidation. LHCII proteins bind to PSII forming the PSII-LHCII complex which in turn forms PSII-LHCII supercomplexes in the thylakoid membrane.

Assembly of PSII in chloroplasts occurs in a stepwise manner (Baena-González and Aro, 2002; Rokka et al., 2005; Mulo et al., 2008; Nixon et al., 2010; Komenda et al., 2012; Lyska et al., 2013; Nickelsen and Rengstl, 2013). First, the precursor of D1 (pD1) is co-translationally assembled into the D2-Cyt *b*_559_ receptor complex to form the PSII RC with the PsbI subunit. The C-terminal 9–13 residues of pD1 are subsequently removed by the CtpA enzyme to form functional D1 protein (Che et al., 2013). Second, CP47 is integrated into the RC to form the CP47-RC complex. The chloroplast-encoded proteins PsbH, PsbM, PsbTc, and the nucleus-encoded protein PsbR are assembled into the CP47-RC complex to form CP43-less PSII. In the next step, CP43 and other subunits are integrated to form the PSII monomer. Finally, the PsbO, PsbP, and PsbQ subunits associate with the PSII monomer and form supercomplexes with LHCII eventually with the help of PsbJ and PsbZ (Rokka et al., 2005).

More than a dozen PSII assembly factors are required for the PSII assembly at various steps in higher plants. For the biogenesis of the PSII RC complex, several facilitating factors are involved in particular steps such as *psbA* translation, pD1 processing, and assembly of the complex. HCF173 is a protein weakly related to short-chain dehydrogenases/reductases. It forms a high molecular weight complex that both facilitates translational initiation of D1 and stabilizes the *psbA* mRNA (Schult et al., 2007). After translation, the nine residue C-terminal tail of pD1 is cleaved by the lumen-localized C-terminal processing protease CtpA (Che et al., 2013). Two One-Helix-Proteins OHP1 and OHP2 as well as the HCF244 protein form a complex essential for the formation of the PSII RC complex. Formation of the OHP1/OHP2/HCF244 complex is likely required for the efficient recruitment of ribosomes with *psbA* mRNA encoding D1 and also for the delivery of chlorophyll and/or other cofactors to the D1/D2 heterodimer (Beck et al., 2017; Hey and Grimm, 2018; Li et al., 2018; Myouga et al., 2018; Chotewutmontri et al., 2020). In addition, thylakoid lumen protein HCF136 is essential for PSII RC formation (Meurer et al., 1998; Plücken et al., 2002). YCF48, the ortholog of HCF136 in cyanobacteria and red algae, is a seven-bladed beta-propeller that promotes efficient incorporation of D1 into the PSII RC complex by interacting with newly synthesized pD1 on the thylakoid lumen side (Komenda et al., 2008; Yu et al., 2018). Chloroplast-encoded PsbN is not a PSII subunit but rather acts as a molecular chaperone required in the early phase of PSII RC formation (Torabi et al., 2014).

Rubredoxins are iron-containing proteins in which one iron atom is coordinated by four cysteine residues, and they participate in electron transfer reactions (Lovenberg and Sobel, 1965; Adman et al., 1975). RBD1 (Rubredoxin 1) from Chlamydomonas and its ortholog from cyanobacteria RubA contain one rubredoxin domain and one transmembrane domain. Loss of RBD1 in photosynthetic eukaryotes impacts the functional accumulation of PSII (Calderon et al., 2013). Further studies demonstrated that they may participate in PSII RC formation by participating together with cyt *b*_559_ to protect PSII assembly intermediates from photooxidation or maintaining PSII co-factors in a suitable redox state to prevent photodamage (García-Cerdán et al., 2019; Kiss et al., 2019).

In this study, we characterized the *rbd1* mutant of Arabidopsis. We demonstrate that ribosome recruitment of *psbA* mRNA encoding the D1 protein is specifically reduced in *rbd1*, suggesting that RBD1 may act as a regulatory factor involving D1 translation. The PSII reaction center accumulates in higher amounts in *rbd1* but further association with other PSII assembly intermediates to form the larger PSII complex and supercomplexes is nearly totally blocked. These data imply that Arabidopsis RBD1, similarly to the orthologs found in Chlamydomonas and cyanobacteria, exerts a role in the formation of a functional PSII core complex during the early stages of PSII assembly.

## Materials and Methods

### Plant Materials and Growth Conditions

The T-DNA insertion mutant *rbd1* (WiscDsLoxHs187_05C) was obtained from Nottingham Arabidopsis Stock Center (NASC). Position of the T-DNA insertion in *rbd1* was confirmed by PCR using primers in Supplementary Table 1 and subsequent sequencing of the PCR products. Wild type (Columbia, Col-0), *rbd1*, and *rbd1*-com plants were grown on Murashige and Skoog (MS) basal medium containing 3% sucrose and 0.05% (w/v) MES (2-morpholinoethanesulfonic acid) (pH 5.8) under a 16-h photoperiod with 50 μmol photons m^–2^ s^–1^ at 23°C in a greenhouse.

For complementation analysis, a genomic DNA fragment containing *RBD1* and its promoter (750 bp upstream of transcription initiation site) was amplified by PCR using primers RBD1-1301-F and RBD1-1301-R (Supplementary Table 1). PCR products were then cloned into the pCAMBIA1301 vector. The resulting vector was then transferred into *Agrobacterium tumefaciens* C58C and transformed into heterozygous *rbd1* plants by the floral dipping method (Clough and Bent, 1998).

### Chlorophyll Fluorescence Analysis

Images of chlorophyll *a* fluorescence were captured with a MAXI-IMAGING-PAM chlorophyll fluorometer (Walz, Germany) with default parameters. Plants were first kept in darkness for 20 min and the minimum fluorescence (F_*o*_) was measured by turning on the measuring light (ML). Maximum fluorescence (F_*m*_) was then determined with a saturating pulse (SP). The maximum quantum yield of PSII was calculated as F_*v*_/F_*m*_ = (F_*m*_-F_*o*_)/F_*m*_. Images of F_*o*_ and F_*v*_/F_*m*_ were displayed using a false color scale from 0 to 1.

Transients of Chlorophyll *a* fluorescence were monitored using a PAM-2500 chlorophyll fluorometer (Walz, Germany). Plants were dark adapted for 20 min. Then F_*o*_ and F_*m*_ were determined with the measuring light (ML, red light, intensity 2) or a saturating pulse (SP, red light, intensity 10, 0.6 s), respectively. Then, the leaves were illuminated with actinic light (AL, red light, 64 μmol photons m^–2^ s^–1^) for 4 min and the steady-state fluorescence (F_*t*_) was recorded.

### Antibody Generation and Resources

The sequence encoding the soluble part of RBD1 protein (amino acid residues 13–174) was amplified by PCR from cDNA with primers of RBD1-ab-F and RBD1-ab-R (Supplementary Table 1). The PCR products were cloned into the pET28a expression vector (Novagen, United States) by double enzyme digestion and the reconstructed vector was transformed into *E. coli* Rosetta competent cells for expression of the recombinant protein. After induction with 0.5 mM IPTG for 4 h at 37°C, the cells were harvested and recombinant protein was purified from the supernatant on a Ni-NTA agarose column (Qiagen). Polyclonal antibody against RBD1 was raised in rabbits.

Antibodies of D1 (PHY0057, 1:2,000), D2 (PHY0060, 1:2,000), CP43 (PHY0318, 1:5,000), CP47 (PHY0319, 1:2,000), PsbE (PHY0506A, 1:5,000), PsbI (PHY0132A, 1:1,000), PsbO (PHY0344, 1:2,000), PsaA (PHY0342, 1:2,000), PsaD (PHY0343, 1:5,000), Cyt *b*_6_ (PHY0020S, 1:1,000), CF_1_γ (PHY0313, 1:1,000), CF_1_ε (PHY0314, 1:1,000), and SBPase (PHY0410S, 1:1,000) were obtained from PhytoAB.

### Isolation of Thylakoid Membranes, BN/2D-PAGE, and Tricine-SDS-PAGE

Chloroplast thylakoids were isolated according to Zhang et al. (2016). Briefly, leaves of 4-week-old plants grown on MS medium were homogenized in isolation buffer I (0.33 M sorbitol, 30 mM Tricine/KOH, pH 8.4, 10 mM NaHCO_3_, 5 mM EGTA, 5 mM EDTA), and the homogenate was filtered through three layers of Miracloth (Calbiochem, United States). After centrifugation at 4,200 *g* for 5 min at 4°C and resuspension in isolation buffer II (0.3 M sorbitol, 20 mM Hepes-KOH, pH 7.6, 5 mM MgCl_2_, 2.5 mM EDTA), intact chloroplasts were osmotically ruptured in lysis buffer (5 mM MgCl_2_, 2.5 mM EDTA, 20 mM Hepes-KOH, pH 7.6). Thylakoid membranes were separated by centrifugation at 10,000 *g* for 2 min at 4°C.

BN-PAGE, SDS-urea-PAGE, and immunoblot analysis were performed as previously described (Zhang et al., 2016). For 2D/SDS-PAGE, excised BN-PAGE strips were incubated with 2 × SDS sample buffer (50 mM Tris–HCl, pH 6.8, 8 M urea, 5% SDS, 20% glycerol, 5% b-mercaptoethanol, 1% bromophenol blue) for 30 min at 25°C and layered onto denaturing 15% SDS-urea-PAGE gels. For Tricine-SDS-PAGE, samples were solubilized in 4 × Tricine sample buffer (12% SDS, 6% β-mercaptoethanol, 30% glycerol, 150 mM Tris–HCl, pH 7.0, 0.05% Coomassie Brilliant Blue G250) and separated on 10/16% step-gradient Tricine-SDS gels. For immunoblot analysis, proteins separated by SDS-urea-PAGE or Tricine-SDS-PAGE were transferred to nitrocellulose membranes (GE Healthcare, United States) and incubated with various antibodies. Signals were captured with a WSE-6200 LuminoGraph II Chemiluminescence Imaging system (ATTO Technology, Japan).

### Nucleic Acid Analysis

Arabidopsis crude DNA was extracted from leaves through shock crushing with DNA extraction buffer (0.2 M Tris–HCl, pH 8.0; 12.5 mM EDTA, 0.25 M NaCl, 0.5% SDS), and the DNA was precipitated with ethanol and centrifuged at 13,500 *g* for 10 min at room temperature. The DNA was solubilized and subjected to PCR analysis. RNA gel blot analysis was performed according to Zhang et al. (2018). Total RNA was extracted from the leaves of 4-week-old plants with liquid nitrogen using TRlzol regent (Invitrogen, United States). Equal amounts of total RNA were separated by formamide denaturing agarose gel electrophoresis and transferred to a nylon membrane (GE Healthcare, United States). RNA was detected using nucleic acid probes labeled with digoxigenin (DIG, Roche, Switzerland).

Polysome association analysis was performed according to our previous studies (Zhang et al., 2018). Leaves of 4-week-old plants were ground in liquid nitrogen, and polysomes were extracted with polysome extraction buffer (0.2 M Tris–HCl, pH 9.0, 0.2 M Sucrose, 0.2 M KCl, 35 mM MgCl_2_, 25 mM EGTA, 1% [v/v] Triton X-100, 2% [v/v] polyoxyethylene-10-tridecyl ether, 0.5 mg mL^–1^ heparin, 0.1 M 2-mercaproethanol, 0.1 mg mL^–1^ chloramphenicol, 25 μg mL^–1^ cycloheximide). After centrifugation at 20,000 *g* for 5 min at 4°C, the supernatants were further solubilized with 0.5% (w/v) sodium deoxycholate. Unresolved membrane was removed by centrifugation at 20,000 *g* for 15 min at 4°C, and the supernatants were subsequently layered on 15–55% sucrose gradients. After centrifugation at 237,000 *g* in the SW55 rotor for 65 min at 4°C, each gradient was divided into 10 0.5-mL fractions and proteins associated with RNA were denatured with 0.5% (w/v) SDS and 20 mM EDTA, and then RNA was extracted with acid phenol/chloroform/isoamyl alcohol (25:24:1). The RNA associated with polysomes was precipitated with 95% ethanol at room temperature and solubilized in DEPC-treated water. The samples were subjected to RNA gel blot analysis.

### *In vivo* Labeling and Chasing of Chloroplast Proteins

Chloroplast proteins labeling and chasing analysis was performed as previously described (Zhang et al., 2018). Labeled proteins were fractionated by SDS-urea-PAGE or 2D BN/SDS-PAGE, and signals were detected by autoradiography.

### Ribosome Profiling and RNA-Seq

The ribosomal profiling analysis was performed by Gene *Denovo* Biotechnology Co., (Guangzhou, China) according to Ingolia et al. (2012) with modifications. Leaves of 4-week-old mutants and WT plants were ground to powder in liquid nitrogen and then dissolved in lysis buffer (20 mM Tris HCl pH 8.0, 1.5 mM MgCl_2_, 140 mM KCl, 100 μg mL^–1^ chloramphenicol, 100 mg mL^–1^ cycloheximide, 1% Triton-X-100). After centrifugation at 20,000 *g* for 10 min at 4°C, the supernatant was treated with RNase I (NEB, United States) and DNase I (NEB, United States) at room temperature for 45 min to degrade DNA and unprotected RNA. Nuclease digestion was stopped by adding RNase inhibitor (SUPERase•In; Ambion, United States). Then, digested RNAs were separated by size exclusion columns (illustra MicroSpin S-400 HR Columns; GE Healthcare) and SDS was added to the elution at a final concentration of 1% to denature RNA-associated proteins. RNAs with a size greater than 17 nt were isolated with RNA Clean and Concentrator-25 kit (Zymo Research, United States). rRNA was removed as reported previously (Morlan et al., 2012), and RNAs were further purified using magnetic beads (Vazyme, China).

Ribo-seq libraries were prepared with NEBNext Multiple Small RNA Library Prep Set for Illumina (NEB, United States) according to the user manual. PCR products with DNA fragments at 140–160 bp (representing insert sizes of 18–35 bp) were enriched to generate a cDNA library and sequenced with Illumina HiSeqTM X10 (Gene *Denovo* Biotechnology Co., China).

Read processing, alignment, and analysis were carried out according to Chotewutmontri and Barkan (2018). Reads counts in the open reading frame (ORF) of coding genes were calculated by software RSEM, and gene expression level was normalized by using RPKM (Reads Per Kilobase of transcript per Million mapped reads) method. Read counts and RPKM values for chloroplast genes are summarized in Supplementary Table 2. Distribution of ribosome footprints along the ORF was normalized by the sum of the total read counts.

The raw data of ribosome profiling reported in this paper have been deposited in the GenBank (NCBI) Sequence Read Archive (SRA) with accession number PRJNA785666^1^.

### Accession Numbers

The sequences data in this assay obtain from the Arabidopsis Information Resource or GenBank/EMBL databases under the following accession numbers: AtRBD1 (AT1G54500, *Arabidopsis thaliana*), CrRBD1 (Cre07.g315150.t1.2, *Chlamydomonas reinhardtii*), and SyRubA (slr2033, *Synechocystis* sp. PCC 6803).

## Results

### The *rbd1* Mutant Is Defective in Growth and PSII Function

**FIGURE 1:**
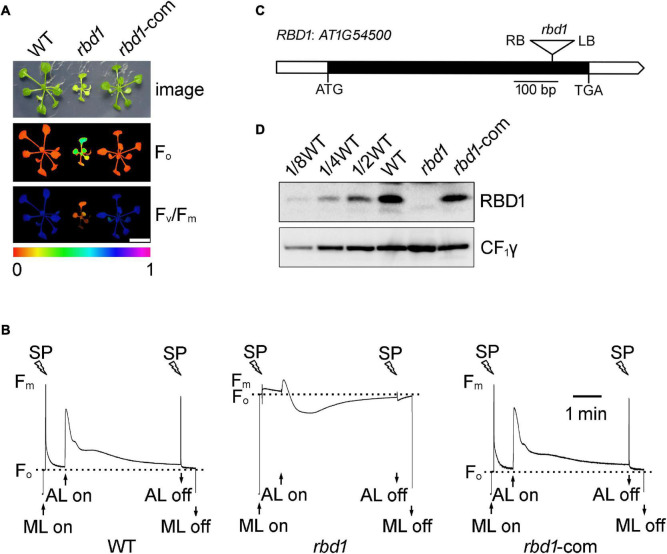
Characterization of the *rbd1* mutant. **(A)** Phenotypes and complementation of the *rbd1* mutant. Growth (upper panel) and chlorophyll *a* fluorescence (F_*o*_, middle panel; F_*v*_/F_*m*_, lower panel) phenotype of wild type (WT), *rbd1*, and *rbd1*-com plants. Values for F_*o*_ and F_*v*_/F_*m*_ are indicated using a false color scale at the bottom. *rbd1*-com, *rbd1* mutant complemented using the RBD1 genomic DNA from WT. **(B)** Chlorophyll *a* fluorescence transients of the *rbd1* mutant. After 20-min dark adaption, minimal fluorescence (F_*o*_) was measured with measuring light (ML) and a saturation pulse (SP) was applied to record maximal fluorescence (F_*m*_). Subsequently, leaves were exposed to actinic light (AL, 64 μmol photons m^–2^ s^–1^) for 4 min. **(C)** Structure of the *RBD1* gene. White and black boxes represent the untranslated and coding regions, respectively. The T-DNA insertion is represented by a triangle. **(D)** Immunoblot analysis of RBD1 protein. Thylakoid proteins extracted from WT, *rbd1*, and *rbd1*-com plants were separated by SDS-urea-PAGE and detected with an antibody against RBD1. CF_1_γ is shown as a loading control.

To investigate the function of RBD1 in higher plants, the T-DNA insertion Arabidopsis mutant *rbd1* was obtained from NASC (Nottingham Arabidopsis Stock Center). As reported previously, the *rbd1* mutant cannot grow photoautotrophically on soil and exhibits a yellow-green leaf phenotype when grown on Murashige and Skoog (MS) medium (Figure 1A, Calderon et al., 2013). Chlorophyll *a* fluorescence imaging and fluorescence transients showed that the minimum chlorophyll *a* fluorescence (F_*o*_) is increased and the variable fluorescence (F_*v*_) decreased in *rbd1*, resulting in a reduced maximum quantum yield of PSII (F_*v*_/F_*m*_) (Figures 1A,B). These results indicate that the PSII activity is reduced in the *rbd1* mutant.

The *RBD1* gene of Arabidopsis does not contain introns (Figure 1C). Sequencing of the PCR products showed that the T-DNA was inserted into the coding region of *RBD1*, resulting in a complete loss of RBD1 protein in thylakoids (Figure 1D). Introduction of the genomic RBD1 gene driven by its own promoter in the *rbd1* mutant completely rescued growth and wild-type chlorophyll *a* fluorescence patterns (Figures 1A,B). Consistently, similar levels of RBD1 protein were detected in WT and complemented plants (Figure 1D). These results demonstrate that loss of the RBD1 protein is responsible for the severe mutant phenotype of *rbd1*.

### The *rbd1* Mutant Accumulates Low Amount of PSII but Relatively High Levels of PSII Assembly Intermediates

**FIGURE 2:**
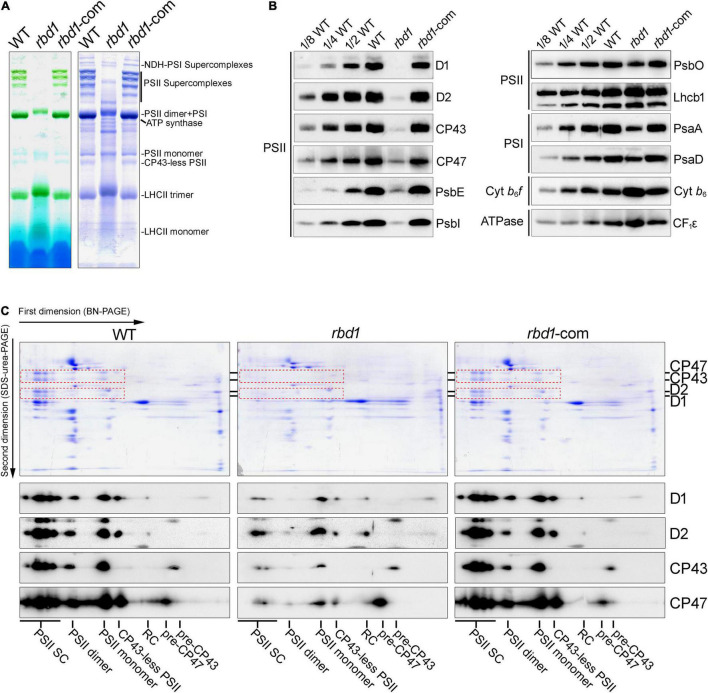
Analysis of thylakoid protein complexes from WT, *rbd1*, and *rbd1*-com plants. **(A)** BN-PAGE analysis of thylakoid membrane protein complexes. Thylakoid protein complexes corresponding to equal amounts of chlorophyll were solubilized with 1% dodecyl-β-D-maltoside (β-DM) and separated by BN-PAGE (left). The gels were stained with Coomassie Brilliant Blue (CBB) (right). **(B)** Immunoblot analysis of representative subunits of thylakoid protein complexes in the *rbd1* mutant. Equal amounts of thylakoid proteins isolated from WT, *rbd1*, and *rbd1*-com plants were separated by SDS-urea-PAGE or Tricine-SDS-PAGE. Immunoblot analysis was performed with specific antibodies indicated on the right side of each panel. A dilution series of the WT sample (1/2, 1/4, and 1/8) was loaded to quantitatively assess protein accumulation in the mutants. **(C)** Two-dimensional BN/SDS-PAGE analysis. For each genotype, a total of five excised BN-PAGE strips **(A)** were incubated with SDS sample buffer for 30 min and layered onto denaturing 15% SDS-urea-PAGE gels. Proteins were stained with CBB (top part) or transferred to nitrocellulose membranes to blot with D1, D2, CP43, and CP47 antibodies, respectively (bottom part). Signals with the same exposure time are shown for each antibody. The spots of D1, D2, CP43, and CP47 on the CBB-stained gels are framed by red dot boxes. PSII-SC, PSII supercomplexes; RC, reaction center.

To examine the effects of RBD1 on the abundance of thylakoid complexes, thylakoids freshly isolated from wild type and *rbd1* mutant were solubilized with 1% *n*-dodecyl β-D-maltoside (β-DM), and protein complexes were separated by blue native polyacrylamide gel electrophoresis (BN-PAGE) (Figure 2A). The results revealed a drastic reduction of PSII complexes including PSII supercomplexes, PSII dimer, PSII monomer, and CP43-less PSII, whereas accumulation of NDH-PSI supercomplex was identical in wild type and *rbd1* mutant (Figure 2A). To quantitate the abundance of PSII subunits in *rbd1*, immunoblotting was performed using WT and *rbd1* thylakoids. Accumulation of the PSII core subunits D1, D2, CP43, CP47, PsbE, and PsbI is significantly reduced to less than 1/8 of WT in *rbd1* (Figure 2B). The level of PSII oxygen-evolving complex subunit PsbO and PSI subunits (PsaA and PsaD) in the *rbd1* mutant is reduced to about 1/2 compared with WT. However, the amounts of LHCII complex (Lhcb1), cytochrome *b*_6_*f* complex (Cyt *b*_6_), and ATP synthase (CF_1_ε) are slightly increased in *rbd1* relative to WT (Figure 2B). Similar levels of these thylakoid membrane proteins were observed in the Chlamydomonas RBD1-deficient mutant *2apc.* The decrease of PSI in *rbd1* is likely a secondary effect of the drastic reduction of PSII (Calderon et al., 2013). These data indicate that, similar to its Chlamydomonas ortholog, Arabidopsis RBD1 is required for the accumulation of PSII complexes (García-Cerdán et al., 2019).

To further investigate the accumulation of the PSII assembly intermediates in *rbd1*, thylakoid protein complexes separated in the first dimension by (1D) BN-PAGE were then resolved into their subunits in the second dimension by (2D) electrophoresis on 15% SDS-urea-PAGE (Figure 2C). Immunoblot analysis showed that the PSII core subunits D1, D2, CP43, and CP47 are present as expected in the PSII supercomplexes (PSII-SC), PSII dimer, PSII monomer, and CP43-less PSII in WT and complemented plants (Figure 2C). However, in the *rbd1* mutant, D1 and D2 are mainly present in the PSII monomer and CP43 and CP47 are mainly present in the PSII monomer and their assembly intermediates (pre-CP43 and pre-CP47) (Figure 2C). Moreover, only trace amounts of D1 and D2 were detected in the PSII reaction center (RC) in WT and complemented plants but a relatively higher level of RC assembly intermediate was found in the *rbd1* mutant (Figure 2C). These results show that *rbd1* accumulates a high amount of PSII assembly intermediates including PSII RC, pre-CP43, and pre-CP47.

### Assembly of PSII Is Impaired in the *rbd1* Mutant

**FIGURE 3:**
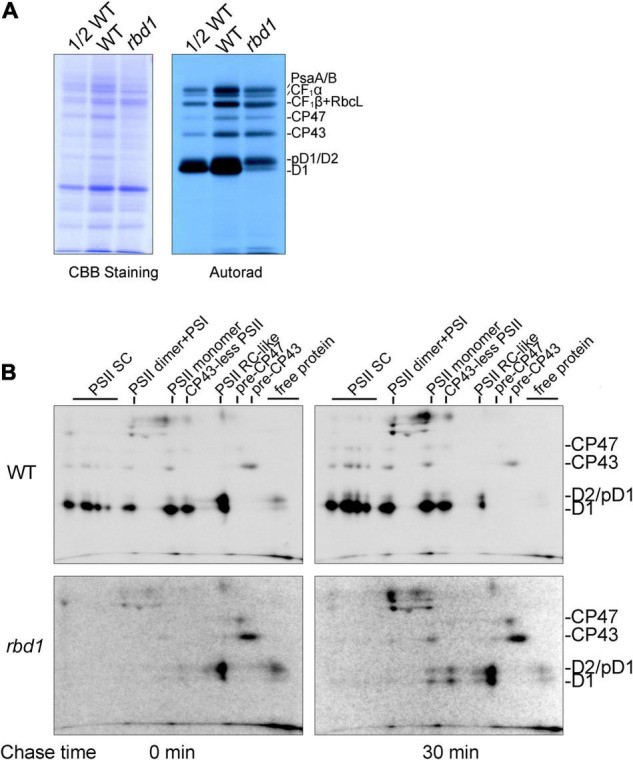
PSII assembly is impaired in the *rbd1* mutant. **(A)** Pulse labeling of thylakoid proteins in the *rbd1* mutant. Primary leaves of 12-day-old plants were labeled with [^35^S]-Met for 20 min in the presence of cycloheximide. Thylakoid proteins were extracted and separated by SDS-urea-PAGE. The gels were stained with Coomassie Brilliant Blue (CBB, left), and labeled proteins were visualized by autoradiography (right). **(B)** Analysis of PSII assembly in *rbd1* mutant. Leaves were labeled as in **A** (indicated as 0 min) and chased with cold Met for 30 min (indicated as 30 min). Thylakoid proteins were isolated and separated by 2D BN/SDS-urea-PAGE. PSII-SC, PSII supercomplexes. Positions of CP47, CP43, D2/pD1, and D1 are shown on the left. The exposure times of autoradiography for WT and *rbd1* are 3 and 15 days, respectively.

To study the assembly kinetics of PSII in the *rbd1* mutant, chloroplast-encoded proteins were labeled with [^35^S]-Met for 20 min using primary leaves of 12-day-old plants in the presence of cyclohexmide which specifically inhibits cytosolic protein synthesis. After labeling, thylakoids were extracted and solubilized in the SDS sample buffer. Equal amounts of protein were separated by 15% SDS-PAGE and the labeled proteins were visualized by autoradiography. As shown in Figure 3A, the most striking difference is that the rate of synthesis of mature D1 protein is drastically reduced in *rbd1* compared with WT (Figure 3A). In contrast, proteins corresponding to pD1 and D2 were heavily labeled in *rbd1* (Figure 3A). As mature D1 is produced from pD1 by removing the C-terminal 9-AA with the CtpA enzyme, it is possible that processing of pD1 protein is retarded in *rbd1*, resulting in a higher accumulation of pD1. Since this band also contains D2 protein, it is possible that the rate of synthesis of D2 is increased in *rbd1* compared with WT.

To distinguish between these two possibilities, we performed pulse-chase labeling experiment. After labeling for 20 min (indicated as 0 in Figure 3B), the labeled proteins were chased with unlabeled Met for 30 min (indicated as 30 in Figure 3B). To investigate the dynamic fates of newly synthesized thylakoid proteins, thylakoid protein complexes were separated by 2D BN/SDS-PAGE and individual subunits were visualized by autoradiography. As shown in Figure 3B, in the WT, mature D1, D2, and CP43 proteins were readily detected in the PSII supercomplexes (PSII SC), PSII dimer, PSII monomer, CP43-less PSII, and PSII reaction center (PSII RC) after labeling for 20 min (indicated as 0 in Figure 3B), indicating efficient PSII assembly. At the PSII RC position, the signal of the protein spot corresponding to pD1/D2 is comparable with mature D1 (Figure 3B). 2D BN/SDS-PAGE of labeled proteins in WT showed that the level of labeled D2 protein is significantly lower than that of D1 in the different PSII complexes. Thus, it is reasonable to speculate that the protein spot corresponding to pD1/D2 in the PSII RC complex represents mainly pD1. As expected, during the 30 min chase, pD1 was rapidly processed to mature D1 protein in the PSII RC complex which was further assembled into various PSII complexes in WT (Figure 3B).

However, after labeling for 20 min, the majority of mature D1 and pD1/D2 was present in the PSII RC and trace amounts of these proteins were in the free form in *rbd1* (Figure 3B). The level of pD1/D2 was significantly higher than mature D1 protein in the PSII RC complex. Even after chasing for 30 min, the majority of D1/pD1 was still present in the PSII RC complex and the level of pD1/D2 was higher than mature D1 in PSII RC (Figure 3B). In addition, CP43 and CP47 were mainly present in the pre-CP43 and pre-CP47 complex, respectively (Figure 3B). These results indicate that PSII assembly of PSII RC complex to CP43-less PSII and other intact PSII complexes is severely impaired in *rbd1*. Since PSII RC contains a high level of mature D1 after chasing for 30 min, it is unlikely that processing of pD1 into D1 represents the main limitation for assembly of PSII in *rbd1*.

Our protein labeling experiments also showed that the rate of synthesis of the PSI core subunits PsaA/B, ATP synthase subunits CF_1_α, as well as the PSII core subunits CP43 and CP47, are slightly reduced in *rbd1* compared with WT (Figure 3A). A similar reduction was also reported for other PSII mutants such as *hcf136*, *hcf244*, *hcf173*, *ohp1*, and *ohp2* (Meurer et al., 1998; Schult et al., 2007; Link et al., 2012; Li et al., 2018) and it is most likely a secondary effect of PSII deficiency.

### Polysome Association With *psbA* Is Reduced in the *rbd1* Mutant

**FIGURE 4:**
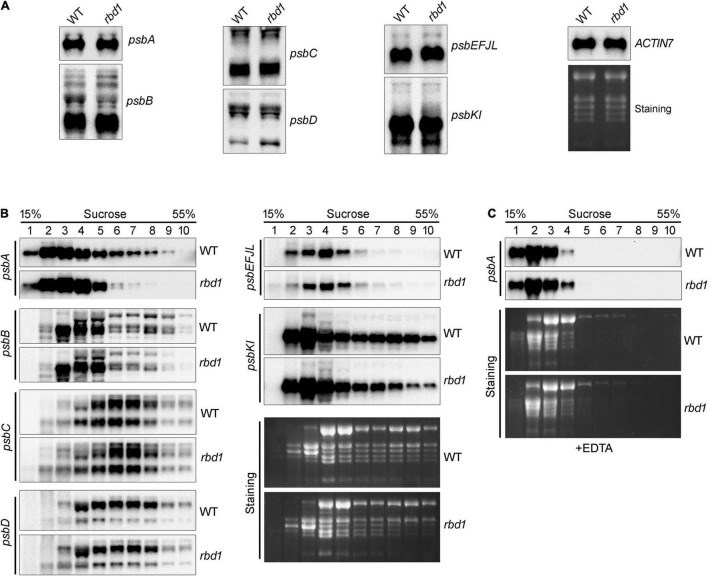
RNA blot analysis of plastid *psb* transcripts encoding PSII subunits and their polysome association. **(A)** RNA blot analysis of the plastid *psb* transcripts encoding PSII subunits. Equal amounts of total RNA extracted with Trizol reagent were separated by electrophoresis on denaturation agarose gels. RNA gel-blot hybridization was performed with DIG-labeled probes specific for *psbA*, *psbB*, *psbC*, *psbD*, *psbEFJL*, *psbKI*, and *ACTIN7*. *ACTIN7* was used as a loading control. **(B)** Polysome analysis. mRNA-polysome complexes isolated from WT and *rbd1* plants were fractionated by 15–55% sucrose density gradient centrifugation. Each gradient was divided into ten 0.5-mL fractions and total RNA was extracted. RNA gel-blot hybridization was then performed with DIG-labeled probes specific for *psbA*, *psbB*, *psbC*, *psbD*, *psbEFJL*, and *psbKI*. RNA staining is shown as a loading control. **(C)** Dissociation of polysomes monitored with a *psbA* probe. Samples were prepared as in **B** except for the addition of EDTA (10 mM) in the polysome extraction buffer. EDTA was used to dissociate polysomes from mRNA.

To investigate why the rate of synthesis of D1 is reduced in *rbd1*, RNA gel blot and polysome association analysis were performed. As shown in Figure 4A, there was no obvious defect in the accumulation and expression pattern of *psbA*, *psbB*, *psbC*, *psbD*, *psbEFJL*, and *psbKI* in *rbd1* compared with WT indicating that RBD1 is not involved in transcription and post-transcriptional processes of the chloroplast *psb* genes. Polysome association analysis which reflects translation initiation of transcripts showed that the distribution of *psbB*, *psbC*, *psbD*, *psbEFJL*, and *psbKI* transcripts was almost identical between *rbd1* and WT (Figure 4B). In contrast, the distribution of *psbA* transcripts was significantly shifted toward the lower molecular weight polysomal fractions in the *rbd1* mutant compared with WT (Figure 4B) indicating that translation initiation of *psbA* transcript is impaired in *rbd1*, resulting in a reduced synthesis rate of D1 *in vivo*. To confirm that the reduction of *psbA* mRNA in the higher molecular weight fractions in *rbd1* was caused by the dissociation with polysomes we performed a control experiment by treating the extract with EDTA before ultracentrifugation. It is known to cause ribosome dissociation from mRNA. As shown in Figure 4C, EDTA treatment induced shifts of *psbA* transcripts towards lower molecular weight fractions in both the *rbd1* mutant and WT, indicating that the amount of polysomes associated with *psbA* transcripts was reduced.

### Ribosome Recruitment to *psbA* mRNA Is Specifically Reduced in *rbd1*

**FIGURE 5:**
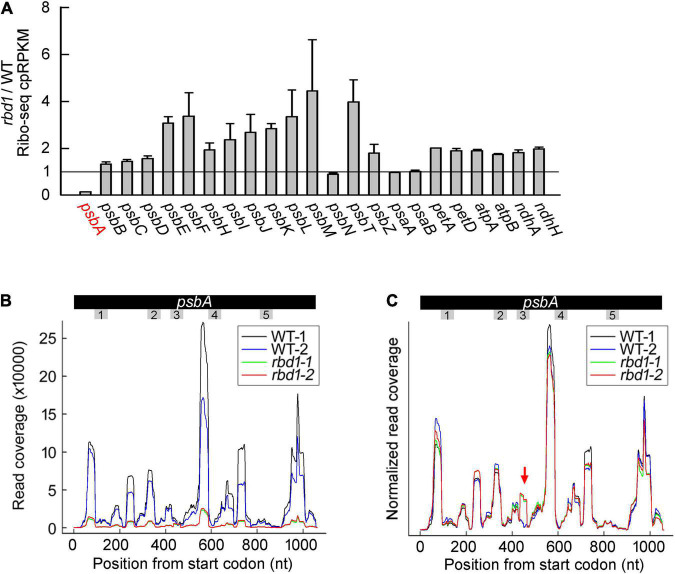
Ribo-seq analysis of the expression of chloroplast gene in the *rbd1* mutant. **(A)** Ribosome footprint abundance for chloroplast genes in *rbd1* relative to wild type. Average of two replicates (mean ± SD) is shown. cpRPKM, reads per kilobase in the ORF per million reads mapped to chloroplast ORFs. **(B)** Distribution of ribosome footprints along the *psbA* ORF in the two replicates of the *rbd1* mutant (*rbd1-1* and *rbd1-2*) and WT (WT-1 and WT-2). The regions of *psbA* mRNA encoding the transmembrane domains (TMD) are labelled from 1 to 5. **(C)** Normalized reads of **(B)**. The *Y*-axis was normalized to the sum of the read coverage. The red arrow points to a new pausing site in *rbd1*.

To investigate whether RBD1 is involved in the translation of other transcripts encoding PSII subunits and other thylakoid complexes, ribosome profiling was performed using WT and *rbd1* plants. In this experiment, ribosome-protected mRNA fragments were deep-sequenced and the reads of each transcript can be calculated by normalizing to total chloroplast reads (cpRPKM). We obtained 63,216,962 and 57,604,852 high-quality clean reads in two replicates for WT and *rbd1*, respectively, after the removal of rRNA, tRNAs, snoRNA, snRNA, miRNA, and low-quality reads (Supplementary Table 2). The proportion of total reads that mapped to the reference genome ranged from 70.43% to 89.39%, and 22,719 genes were identified. As shown in Figure 5A, *psbA* mRNA abundance of ribosome occupancy was drastically reduced to about 14% of WT in *rbd1*, consistent with the lower abundance of polysome association of *psbA* mRNA in the mutant (Figure 4B and Supplementary Table 2). The abundance of ribosome footprints for *psbN*, and *psaA* and *psaB* mRNAs encoding PSI subunits were comparable in *rbd1* and WT. The ribosome footprint abundance of other chloroplast transcripts varied between 1.5- and 4-fold increase compared to WT (Figure 5A). Similar results were also reported for the PSII mutants of *hcf244*, *ohp1*, and *ohp2* (Chotewutmontri et al., 2020). They can be explained by the fact that *psbA* ribosome footprints represent a high fraction of the total footprints in WT and the drastic decrease in *psbA* reads in the mutant therefore increases the cpRPKM values for other genes. If one takes into account this compensation effect, the relatively low *rbd1*/WT cpRPKM ratios for *psbN*, *psaA*, and *psaB* suggest that translation of these proteins may be slightly affected. PsbN is not a PSII subunit but it is involved in the PSII RC formation (Torabi et al., 2014). These facts imply that RBD1 and PsbN share some biochemical or genetic interaction during PSII RC biogenesis.

To further investigate the role of RBD1 in *psbA* mRNA translation, we analyzed the distribution of ribosome footprints along the *psbA* mRNA. Several major peaks were detected both in the mutant and wild type that represent the positions of ribosomes paused temporarily along the mRNA during translation. As expected, the signals of *rbd1* detected in the distribution of *psbA* mRNA were much lower compared to WT because of the lower abundance of ribosome association in *rbd1* (Figure 5B). To facilitate comparison of the ribosome distribution along the *psbA* mRNA in WT and *rbd1*, the Y-axis was adjusted as previously reported (Chotewutmontri et al., 2020). As shown in Figure 5C, the pattern of the ribosome distribution along the *psbA* mRNA in *rbd1* was similar to wild type (Figure 5C). However, a peak was specifically detected in *rbd1* in the region of *psbA* mRNA encoding the latter part of the third transmembrane domain (TMD) (Figure 5C). These results suggest that ribosomes pause during *psbA* translation in *rbd1* when the third TMD of D1 emerges from the ribosomal tunnel. It is thus likely that integration of the first two, probably also the third, TMDs of D1 is retarded during PSII biogenesis in *rbd1*. RBD1 may, directly or indirectly, facilitate the insertion of D1 into thylakoids to form the functional PSII RC complex during assembly and repair of PSII.

## Discussion

Our results show that the levels of the PSII core subunits D1, D2, CP43, and CP47 are reduced to less than 1/8 of WT resulting in a drastic decrease of the various intact PSII complexes as well as PSII activity (Figures 1, 2). Similar phenotypes were also found for the Chlamydomonas *rbd1* and *Synechocystis rubA* deletion mutant (García-Cerdán et al., 2019; Kiss et al., 2019). However, in the Arabidopsis *rbd1* mutant, the steady-state levels of PSII assembly intermediates including PSII RC, pre-CP43, and pre-CP47 complexes are relatively higher compared to WT and the complemented plants (Figure 2C). Consistent with these results, pulse labeling and pulse-chase experiments showed that newly synthesized PSII core subunits are mainly present in the PSII RC, pre-CP43, and pre-CP47 intermediates after labeling for 20 min and an additional 30-min chase (Figure 3B). These results confirm that RBD1 is required for PSII assembly *in vivo* (García-Cerdán et al., 2019; Kiss et al., 2019).

Although the rate of synthesis of pD1 and D1 is reduced in *rbd1*, newly synthesized pD1/D1/D2 proteins are able to assemble into the PSII RC complex in this mutant (Figure 3). However, formation of the larger PSII complexes is severely decreased (Figure 3B). In higher plants, after the formation of the PSII RC complex, CP47 and several low molecular mass subunits (LMM) attach to RC forming CP43-less PSII. Subsequently, CP43 and other LMM PSII subunits are assembled to form the PSII monomer (Rokka et al., 2005). In *rbd1*, newly synthesized CP47 and CP43 are mainly present in the pre-CP47 and pre-CP43 assembly intermediates, respectively (Figure 3B). Pulse labeling showed that the rate of synthesis of CP47 and CP43 is slightly reduced in *rbd1* comparable with WT (Figure 3A). Also, ribosome occupancy of *psbB* and *psbC* mRNA is not reduced in *rbd1* (Figure 5A). These results suggest that newly synthesized CP47 and CP43 are functional and available for further association with the PSII RC complex. However, they are unable to sequentially associate with PSII RC during the labeling and chase period of 50 min (Figure 3B). Hence, it is reasonable to propose that the newly formed PSII RC complex is largely non-functional and therefore cannot bind antennas CP47 and CP43 to form functional PSII. However, we could not exclude the possibility that PSII RC is functional, but the CP47 and CP43 antennas do not have access to PSII RC in the *rbd1* mutant.

In the *Synechocystis rubA* deletion mutant, however, the rates of synthesis of D1 as well as those of the other core subunits D2, CP43, and CP47 are as in WT (Kiss et al., 2019). Moreover, the majority of newly synthesized D1 and D2 remained in an unassembled state in thylakoids and could not assemble into PSII RC complexes as in the Arabidopsis *rbd1* mutant (Figure 3B; Kiss et al., 2019). Since RBD1 in chloroplasts and its cyanobacterial ortholog RubA have conserved rubredoxin and transmembrane domains, it is reasonable to assume that they exert a similar physiological function during PSII assembly. How can one explain the differences between these two species? One explanation could be that the PSII assembly process in cyanobacteria is slightly different. The Arabidopsis *pam68-2* mutant accumulates ∼1/10 level of PSII complex compared with WT but in the *Synechocystis sll0933*, in which the PAM68 cyanobacterial ortholog Sll0933 is disrupted, the PSII complex accumulates almost normally (Armbruster et al., 2010). Further analysis showed that a high level of PSII RC complex is present in *pam68-2* but almost undetectable in the *sll0933* mutant (Armbruster et al., 2010). Similar phenotypes with different levels of PSII assembly intermediates were also found between the *hcf136* Arabidopsis and *ycf48* cyanobacterial mutant (Meurer et al., 1998; Komenda et al., 2008). Moreover, several specific chloroplast PSII assembly factors are absent in cyanobacteria (Nickelsen and Rengstl, 2013). Reciprocally, cyanobacteria also possess specific PSII assembly factors that are missing in chloroplasts (Nickelsen and Rengstl, 2013). These differences in the PSII assembly process and assembly factors may be the result of different environmental living pressures during evolution. As discussed by Nixon et al. (2010), it is also possible that a more effective protein quality system in chloroplasts may lead to differences in accumulation of PSII assembly intermediates in the mutants mentioned above.

How does RBD1 facilitate formation of the functional PSII RC complex? Our protein labeling experiments show that PSII RC containing mature D1 is produced implying that newly synthesized pD1 protein can be properly inserted into thylakoids in the correct topology for processing by the CtpA enzyme in the lumen. RBD1 contains a redox-active rubredoxin domain with a redox midpoint potential of +114 mV that is capable to transfer electrons to cytochrome *c in vitro* (Calderon et al., 2013). Thus, formation of inactive PSII RC in *rbd1* is most likely due to deficient redox control. It is known that D1 integrates co-translationally into the PSII complex during its assembly (Zhang et al., 1999). Addition of DTT at a low concentration partially activated translation elongation of D1 protein pointing to the existence of a redox-sensitive regulatory component (Zhang et al., 2000). It has also been reported that two Cys residues of the cyanobacterial ortholog RubA are reversibly oxidized upon a light-to-dark transition (Guo et al., 2014). It is interesting that one of the two Cys residues (Cys-125) in D1 is positioned at the end of the second transmembrane domain (another one is in the middle of the first loop of D1). Therefore, RBD1 might be the redox regulatory component during the biogenesis of pD1, especially at early stages, when ribosomes pause after the first two transmembrane domains have emerged from the ribosomal tunnel in *rbd1* (Figure 5C).

An alternative possibility for the function of RBD1 is delivery co-factors into PSII RC, as discussed previously (Kiss et al., 2019). The D1/D2 heterodimer binds several redox components including six chlorophyll *a*, two pheophytin (Pheno) *a*, one non-heme iron, as well as quinones Q_*A*_ and Q_*B*_ (Umena et al., 2011). Non-pigmented forms of most chlorophyll-binding proteins are rapidly degraded, suggesting that these co-factors are incorporated into PSII RC during the biogenesis of D1/D2 (Funk et al., 1995). Thus, RBD1 might also facilitate the binding of redox components to D1, such as pheophytin which binds to the residue Glu-130 of D1 at the end of the second transmembrane segment (Giorgi et al., 1996; Sharma et al., 1997). Moreover, it has been shown that iron can be released from the active site of rubredoxin protein under certain conditions (Zheng et al., 2013). Hence, another possibility could be that RBD1 directly delivers the non-heme Fe to the D1/D2 heterodimer.

Our polysome association and ribosome profiling analysis revealed that ribosome occupancy on *psbA* mRNA is drastically reduced in the absence of RBD1 (Figure 5A). A similar decrease was also found in the *hcf244*, *ohp1*, and *ohp2* mutants (Chotewutmontri et al., 2020). These results imply that RBD1 and HCF244/OHP1/OHP2 are involved in the translation initiation of D1. However, a peak downstream of the region encoding the latter part of the third transmembrane domain of D1 is relatively enhanced in *rbd1* compared to WT (Figures 5B,C), indicating that ribosomes pause in *rbd1* after the second transmembrane domain of D1 is released from the ribosomal tunnel. It is possible that, in the absence of RBD1, the first two transmembrane domains cannot properly insert in the D2-Cyt *b*_559_ precomplex because of unavailability for the redox-active cofactors, resulting in ribosome pausing on *psbA* mRNA, and subsequently in the degradation of this ribosome-nascent chain complex, ultimately leading to a decrease of *psbA* mRNA translation (Figures 3, 5). A similar scenario may be also valid for the HCF244/OHP1/OHP2 proteins, which were proposed to deliver chlorophyll to PSII RC (Hey and Grimm, 2018; Li et al., 2018; Myouga et al., 2018; Chotewutmontri et al., 2020). In this case, reduction of polysome occupancy on the *psbA* mRNA is likely a secondary effect of membrane integration deficiency of D1.

In summary, we provide evidence that RBD1 is required for the formation of the functional PSII core complex and probably also required for the translation of D1 during the assembly of PSII at early stages in Arabidopsis. Together with the reports on RBD1 from Chlamydomonas and its ortholog from cyanobacteria RubA (García-Cerdán et al., 2019; Kiss et al., 2019), it is likely that RBD1 orthologs exert a similar role for forming a functional PSII among various photosynthetic organisms.

## One-Sentence Summary

Thylakoid intrinsic protein RBD1 is required for assembly of PSII core complex at early stages.

The author responsible for the distribution of materials integral to the findings presented in this article in accordance with the policy described in the Instructions for Author is: LZ (zhanglin2017@shnu.edu.cn).

## Data Availability Statement

The original contributions presented in the study are publicly available. These data can be found here: National Center for Biotechnology Information (NCBI) BioProject database under accession number PRJNA785666.

## Author Contributions

LC, HM, and LZ conceived the study and designed the experiments. LC, HM, and JR performed the experiments. HM and LZ produced the figures. LC, HM, LP, and LZ wrote the manuscript. LZ supervised the whole study. All authors analyzed the data.

## Conflict of Interest

The authors declare that the research was conducted in the absence of any commercial or financial relationships that could be construed as a potential conflict of interest.

## Publisher’s Note

All claims expressed in this article are solely those of the authors and do not necessarily represent those of their affiliated organizations, or those of the publisher, the editors and the reviewers. Any product that may be evaluated in this article, or claim that may be made by its manufacturer, is not guaranteed or endorsed by the publisher.

## Footnotes

1
https://www.ncbi.nlm.nih.gov/sra/PRJNA785666
