# Supplementary material for: Rubredoxin 1 Is Required for Formation of the Functional Photosystem II Core Complex in Arabidopsis thaliana
Source: Front Plant Sci. 2022 Feb 23;13:824358. doi: 10.3389/fpls.2022.824358 (PMC8905225; doi:10.3389/fpls.2022.824358)
Supplement: Supplementary file 1 [file Data_Sheet_1.PDF]

Supplemental Table S1. Primers used in this work.

Primer names	Sequences 5' to 3'
**Primers used for detection of T-DNA insertion sites**	
LB4	TGATCCATGTAGATTTCCCGGACATGAAG
RBD1-T-DNA-F	TCCTTCTGTCCCCTCTCTCAATC
RBD1-T-DNA-R	GGCAATTGGATGTTATTCGACTC
**Primers used for complementation vector construction**	
RBD1-1301-F	GTTTCTAGAGAGATACCACATGTGGGTCTC
RBD1-1301-R	AACGTCGACTCATTGAATGAAATAGCCACTC
**Primers used for construction of antibody**	
RBD1-ab-F	CCTGAATTCCAATCCCATCCCCATTCTTCTC
RBD1-ab-R	GGTCTCGAGAGTCTTCTGACCAGAGGTAAGAGC
