# Supplementary material for: Rubredoxin 1 Is Required for Formation of the Functional Photosystem II Core Complex in Arabidopsis thaliana
Source: Front Plant Sci. 2022 Feb 23;13:824358. doi: 10.3389/fpls.2022.824358 (PMC8905225; doi:10.3389/fpls.2022.824358)

Figure 1A

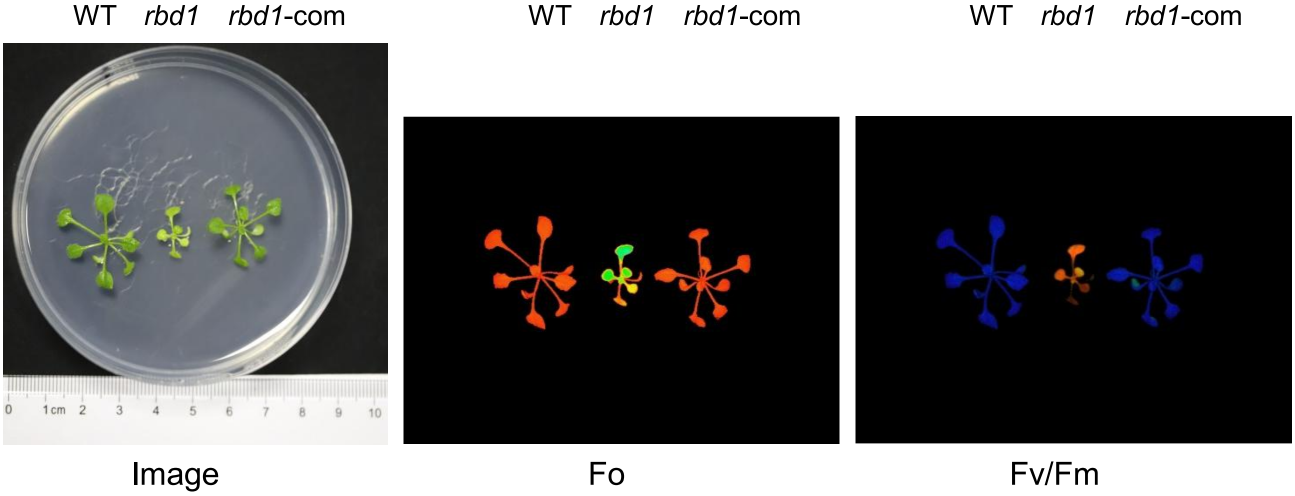

Figure 1D

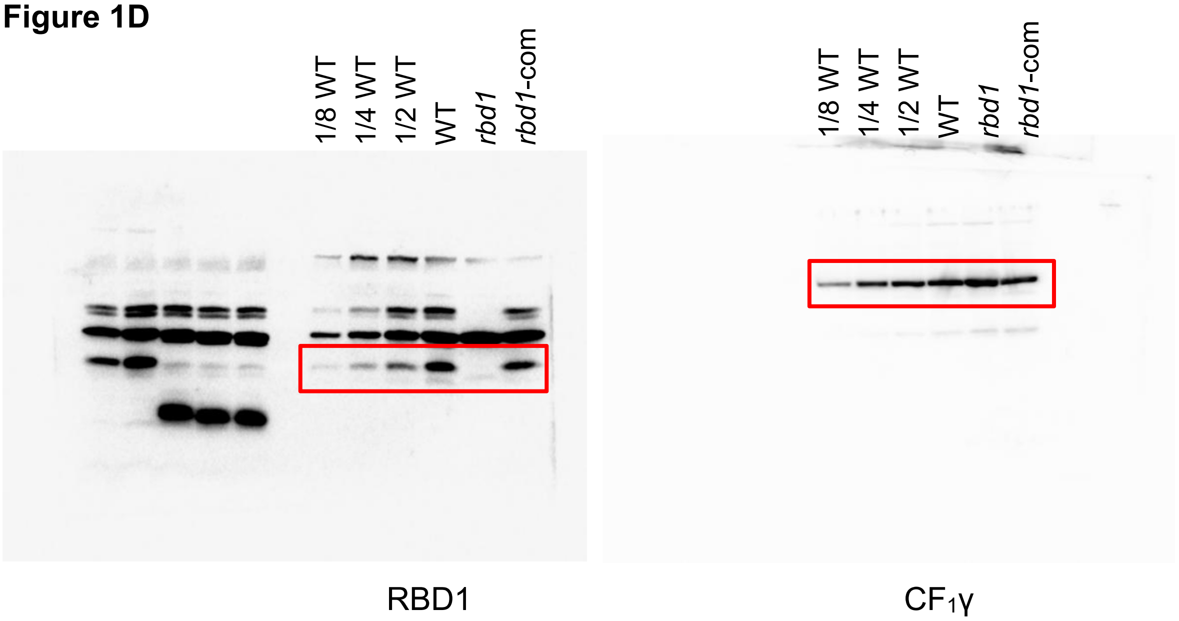

Figure 2A

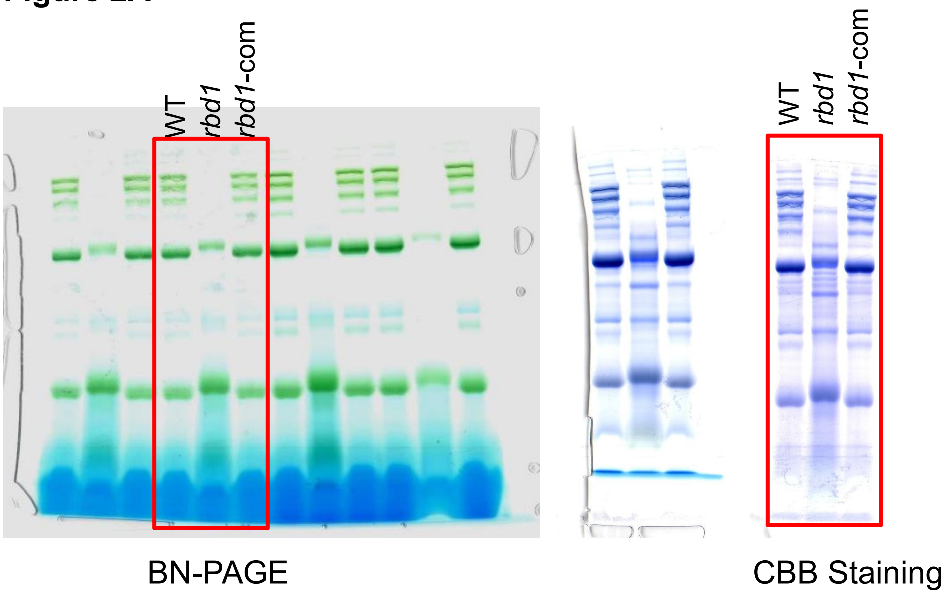

Figure 2B

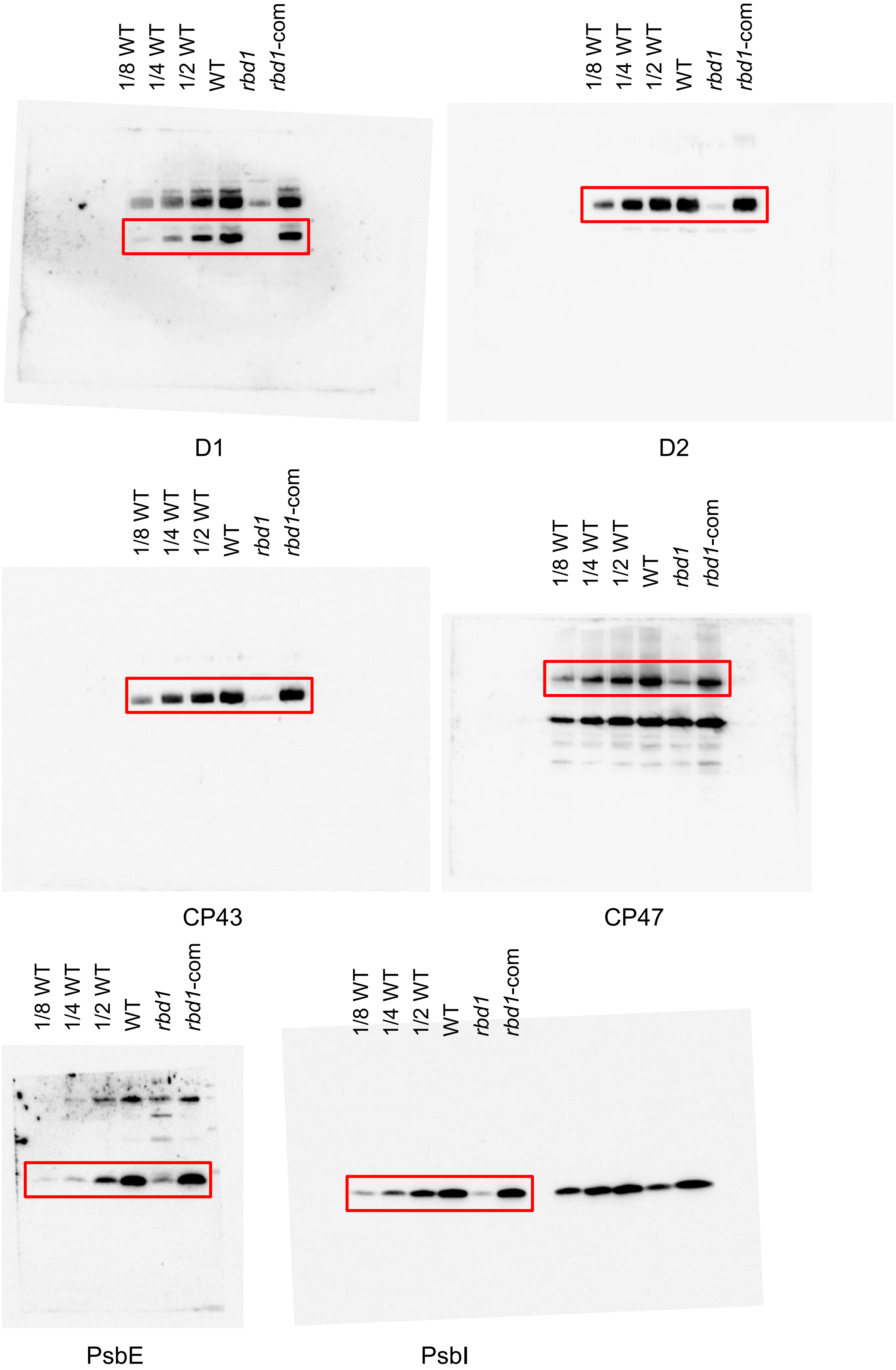

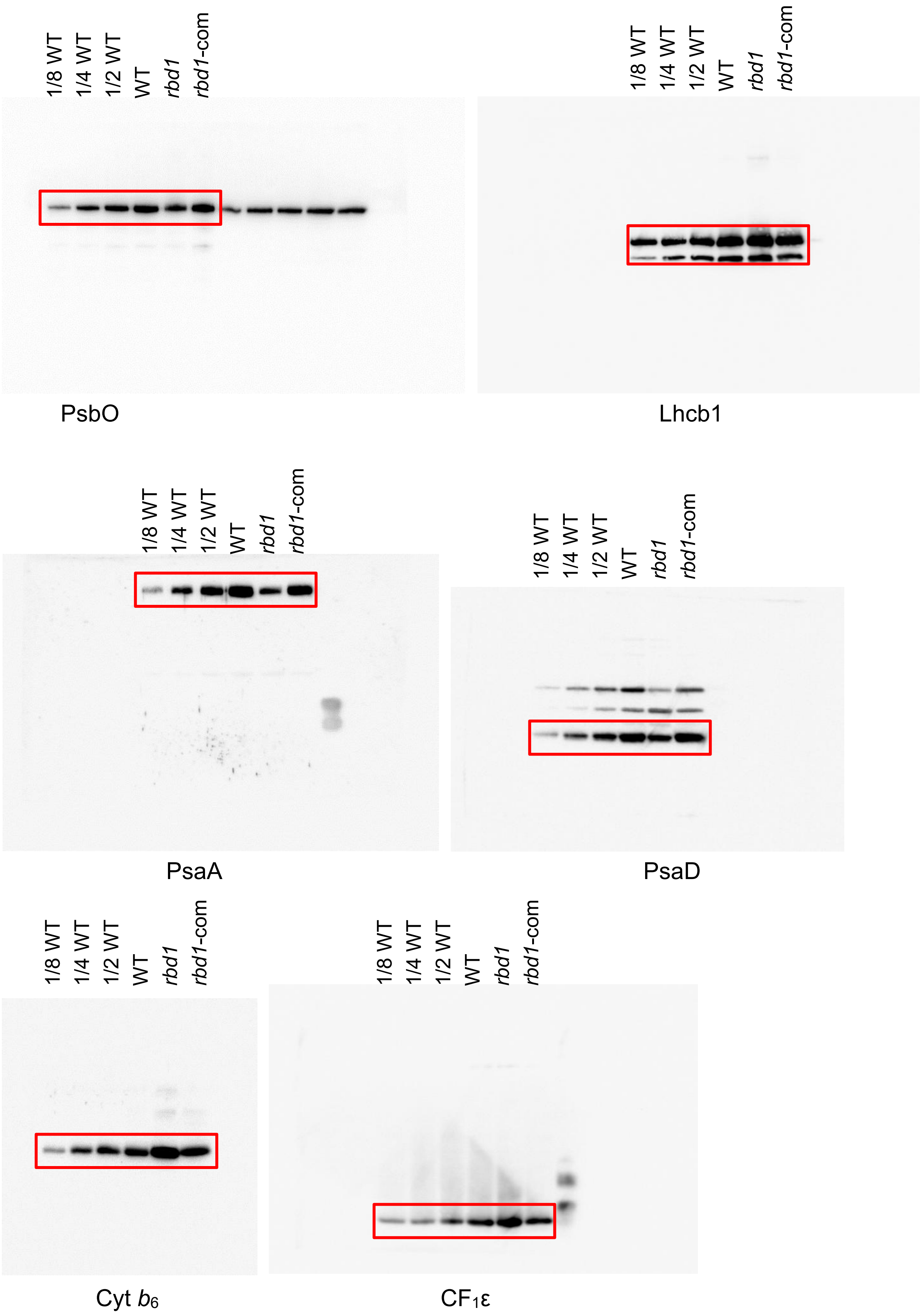
1/8 WT
1/4 WT
1/2 WT
WT
rbd1
rbd1-com
PsbO
Lhcb1
PsaA
PsaD
Cyt b6
CF1ε

Figure 2C

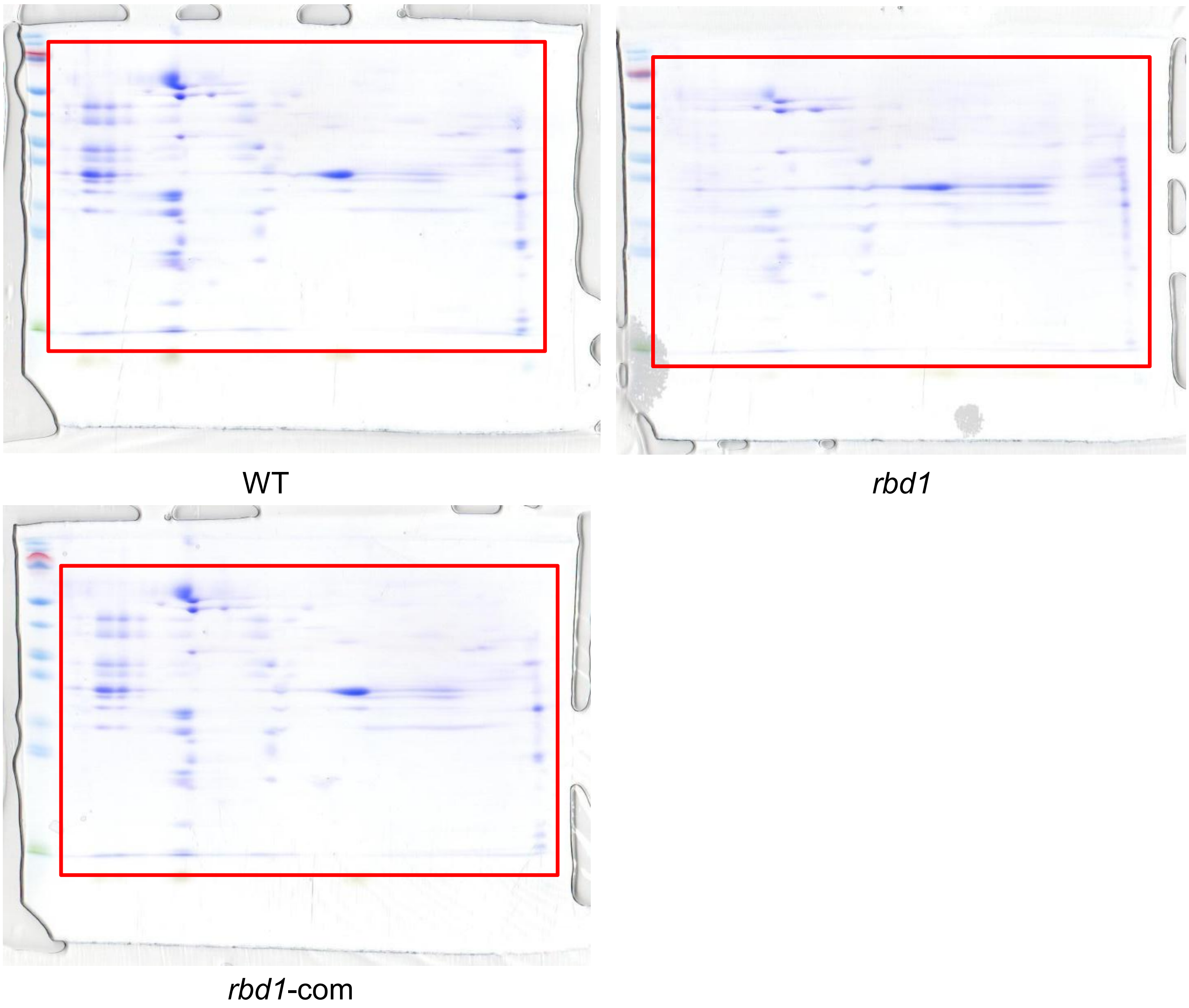

D1

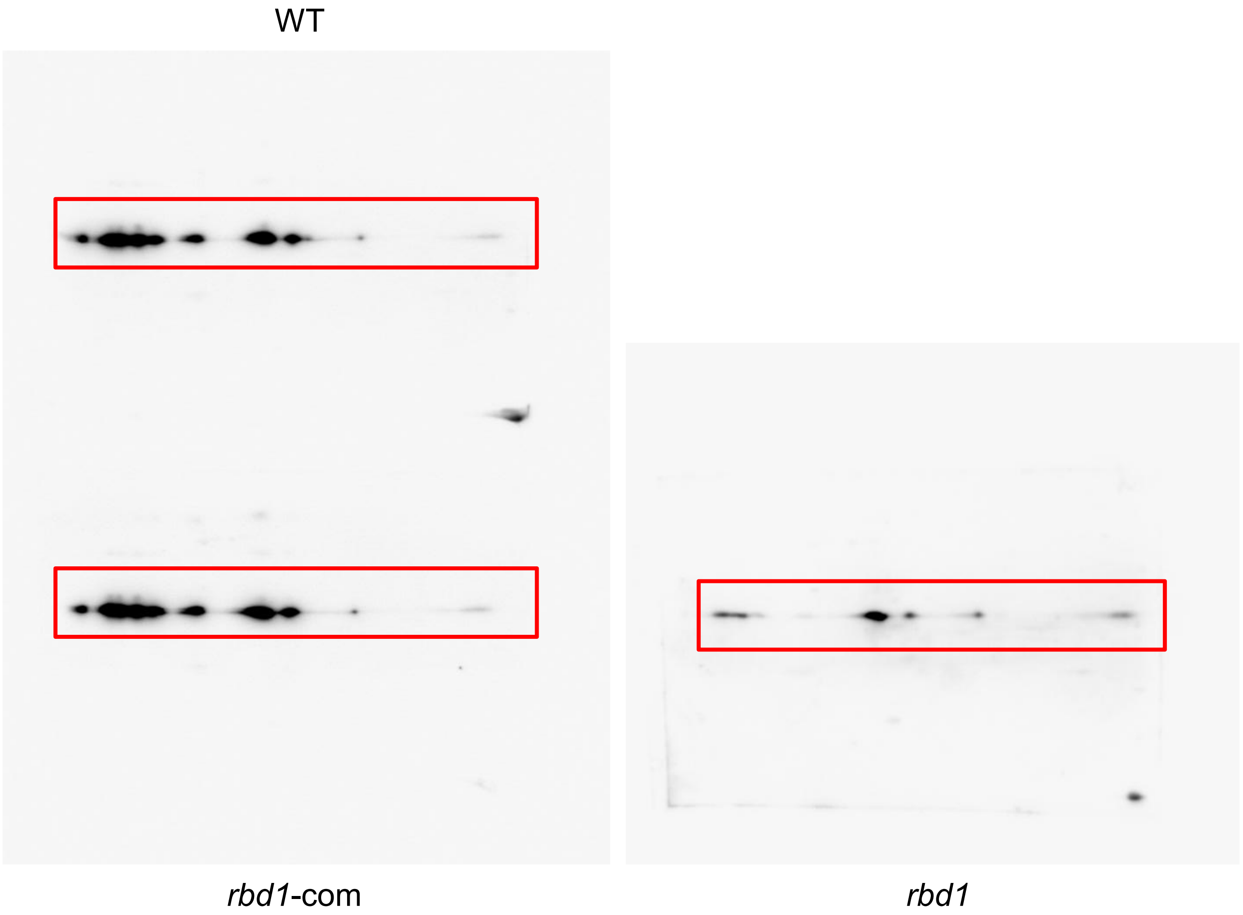

D2

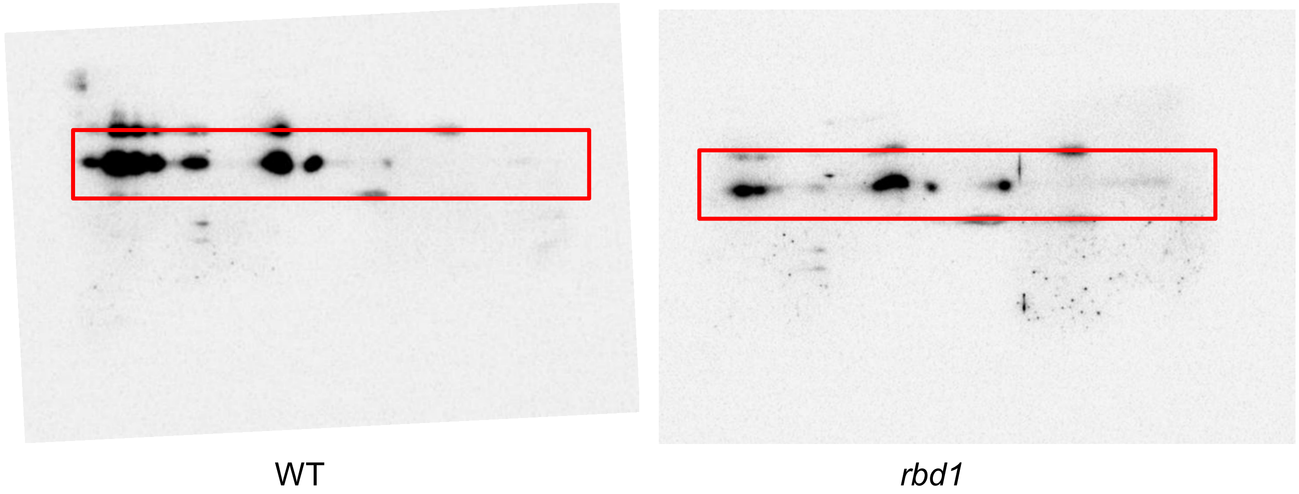

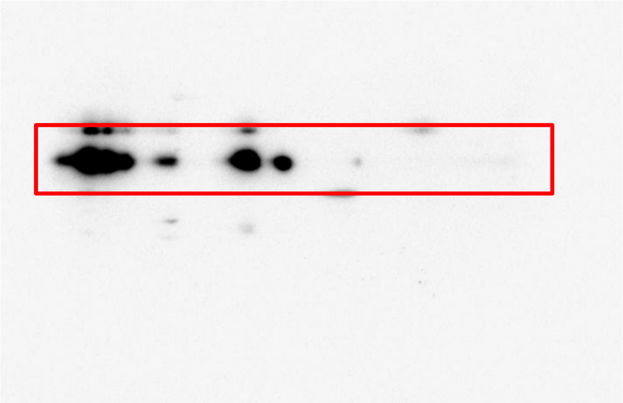

rbd1-com

CP43

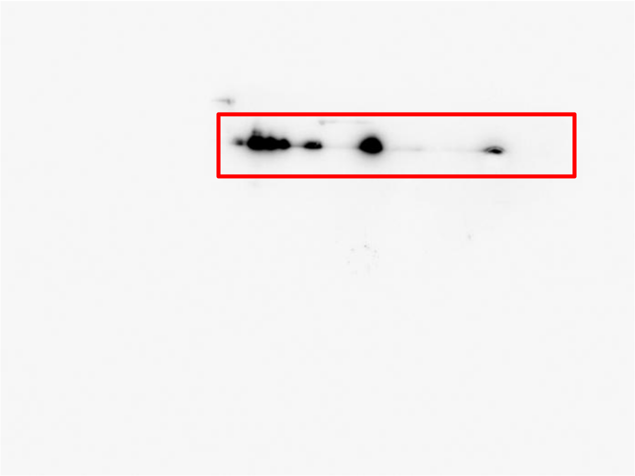

WT

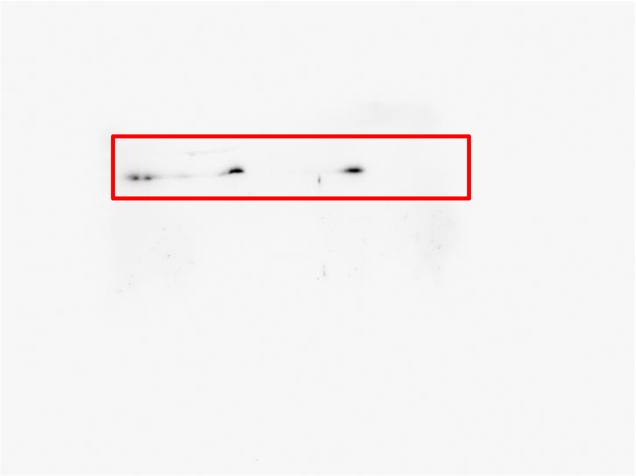

rbd1

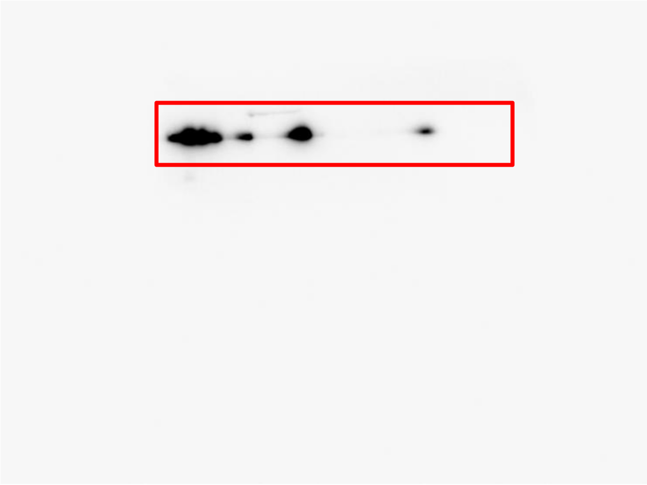

rbd1-com

CP47

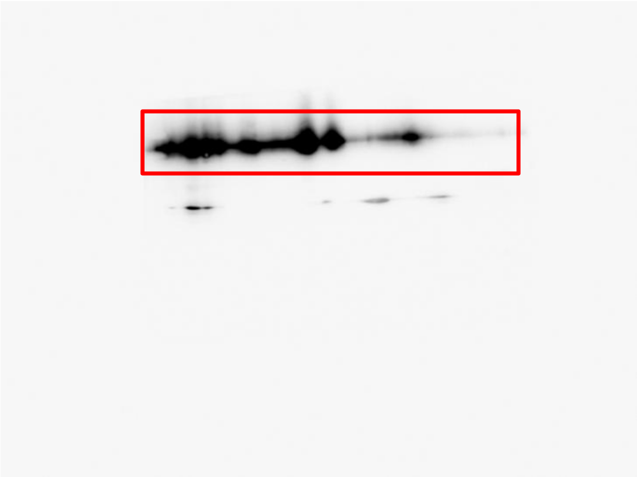

WT

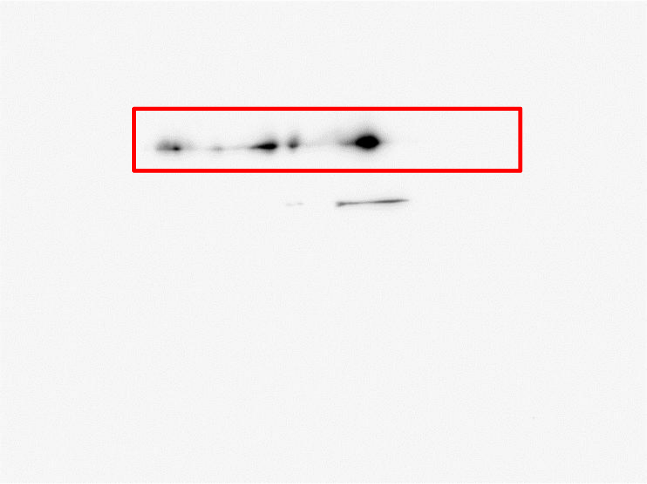

rbd1

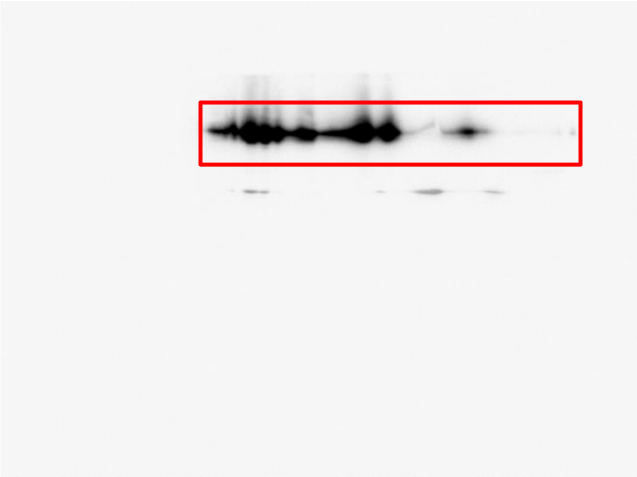

rbd1-com

Figure 3A

1/2 WT
WT
rbd1

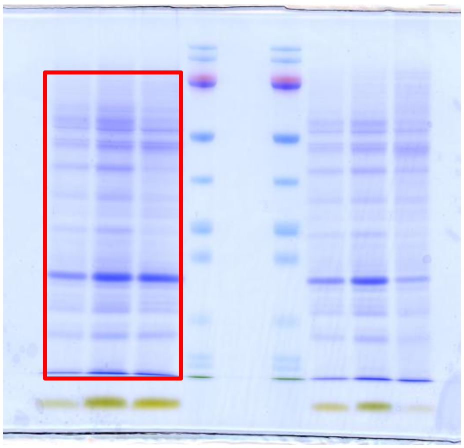

CBB Staining

1/2 WT
WT
rbd1

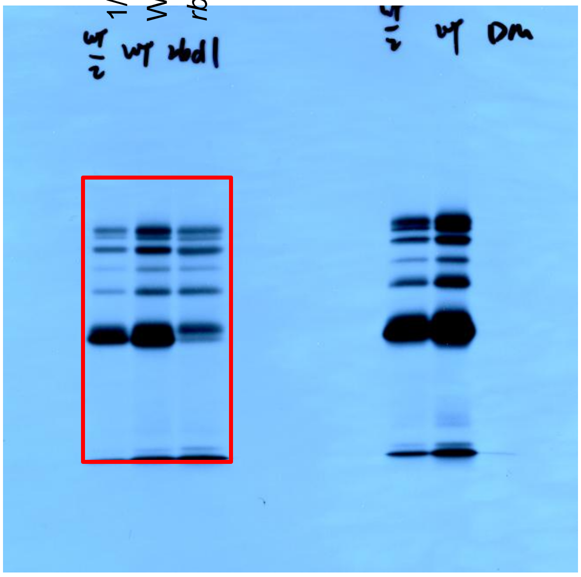

Autorad

Figure 3B

WT

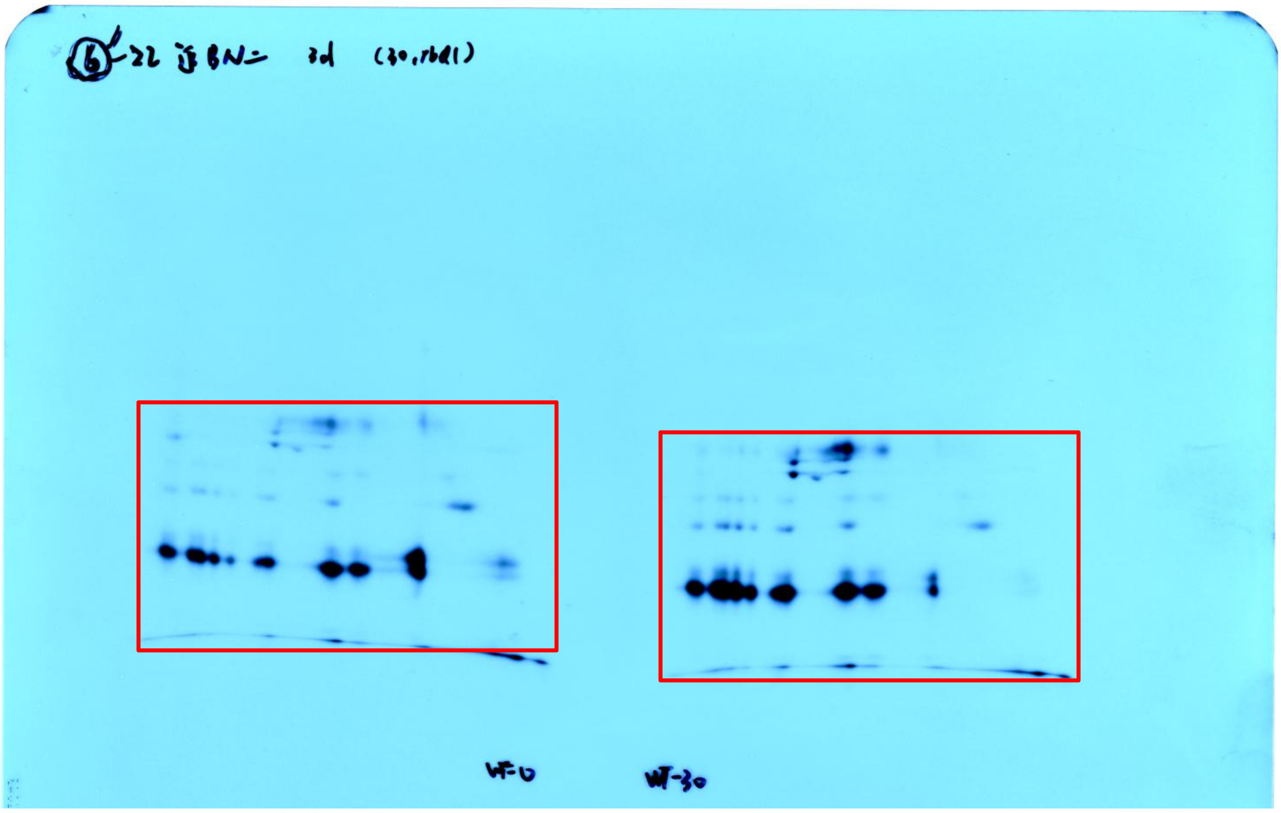

0 min 30 min

rbd1

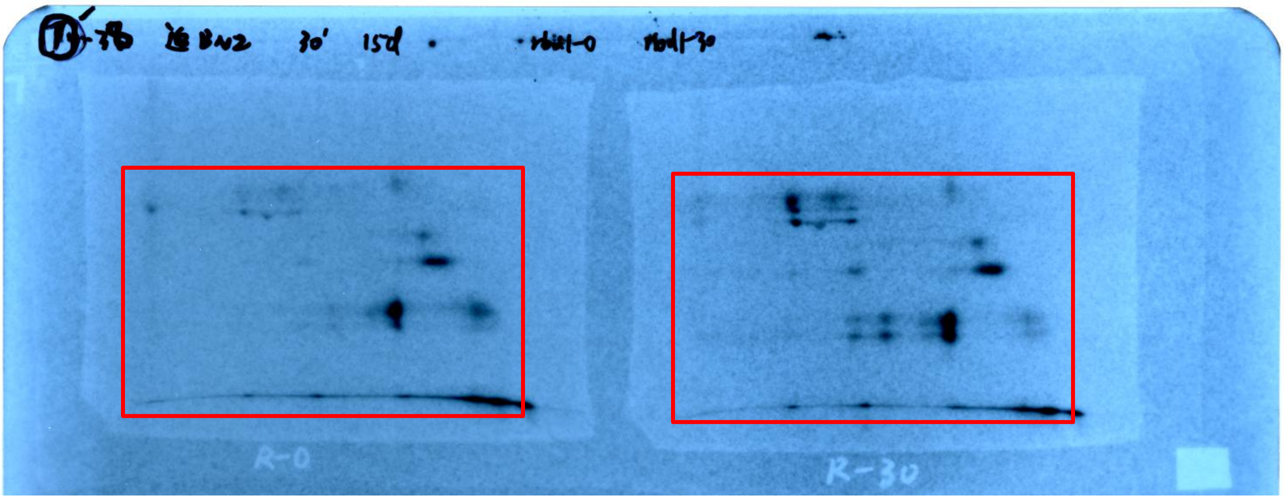

0 min 30 min

Figure 4A

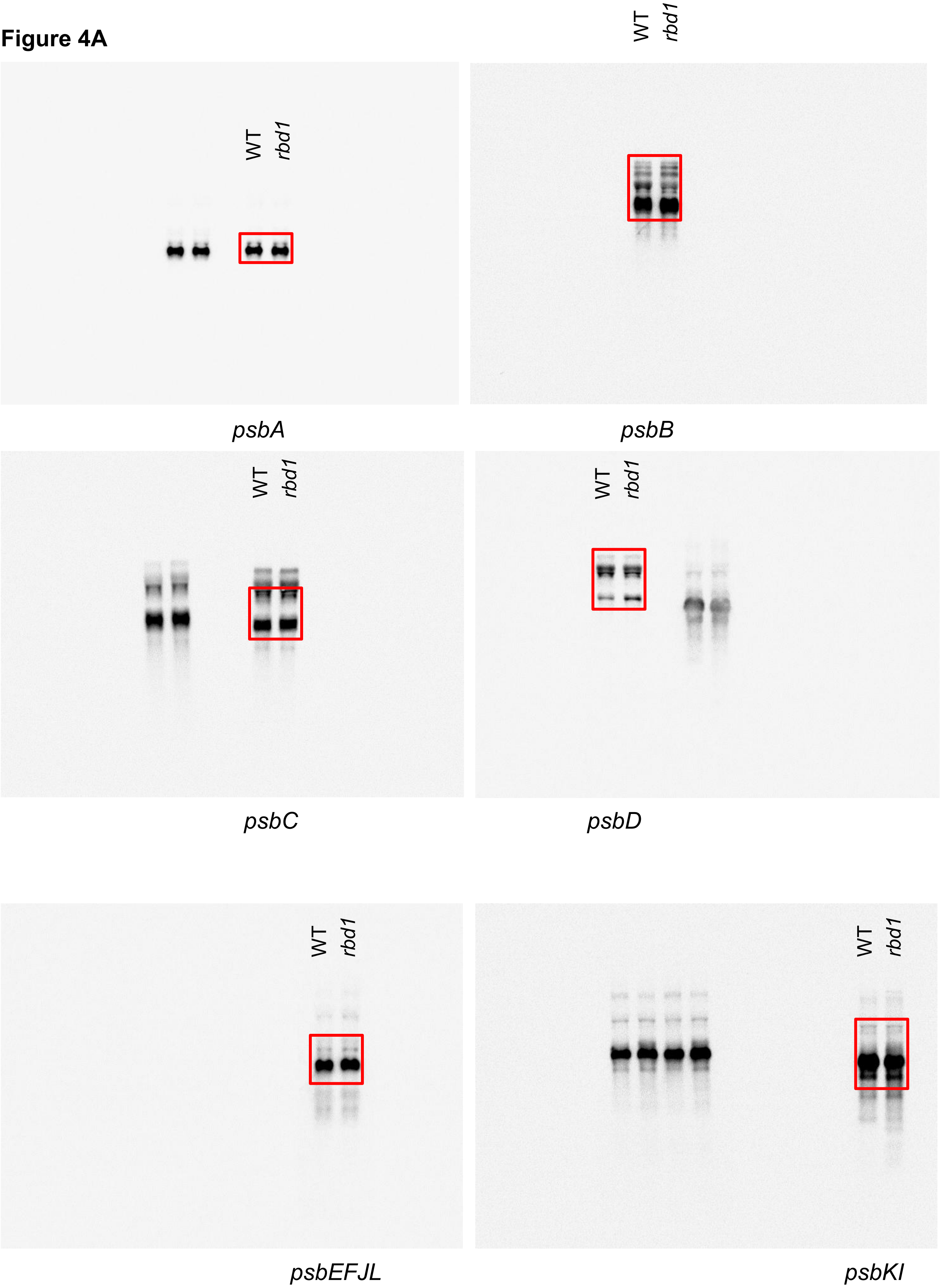

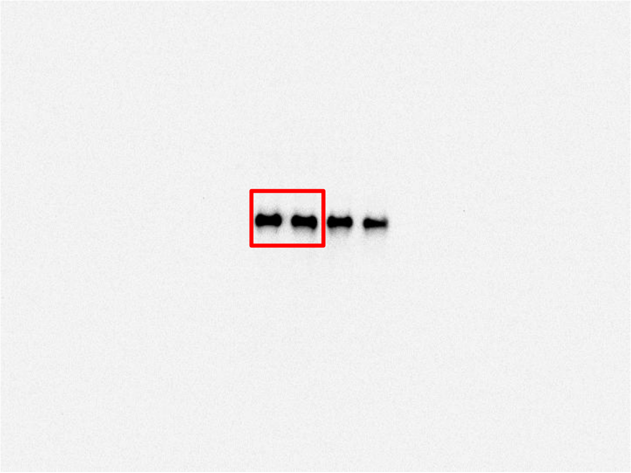

ACTIN7

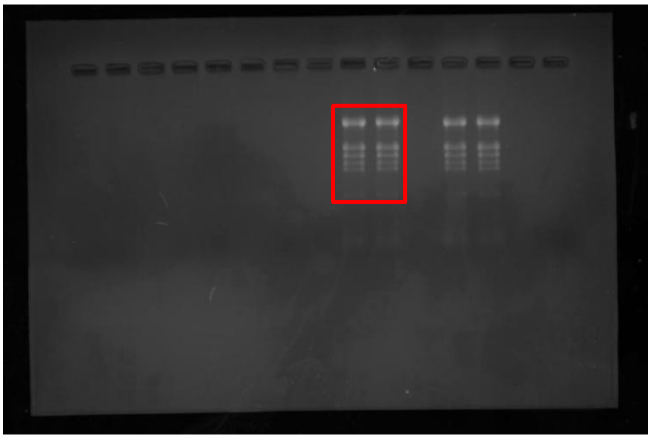

Staining

Figure 4B
psbA

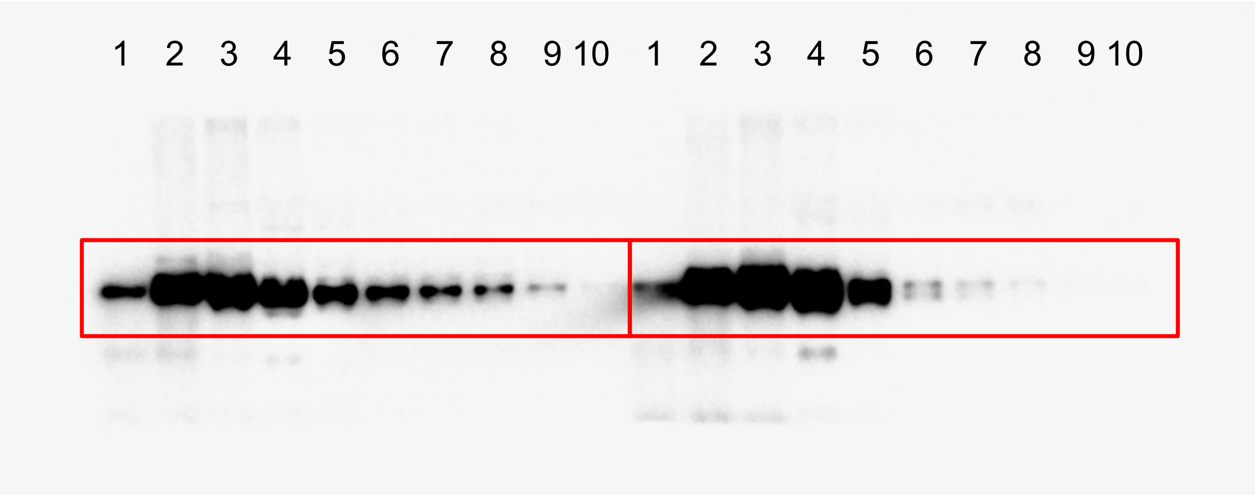

WT *rbd1*

psbB

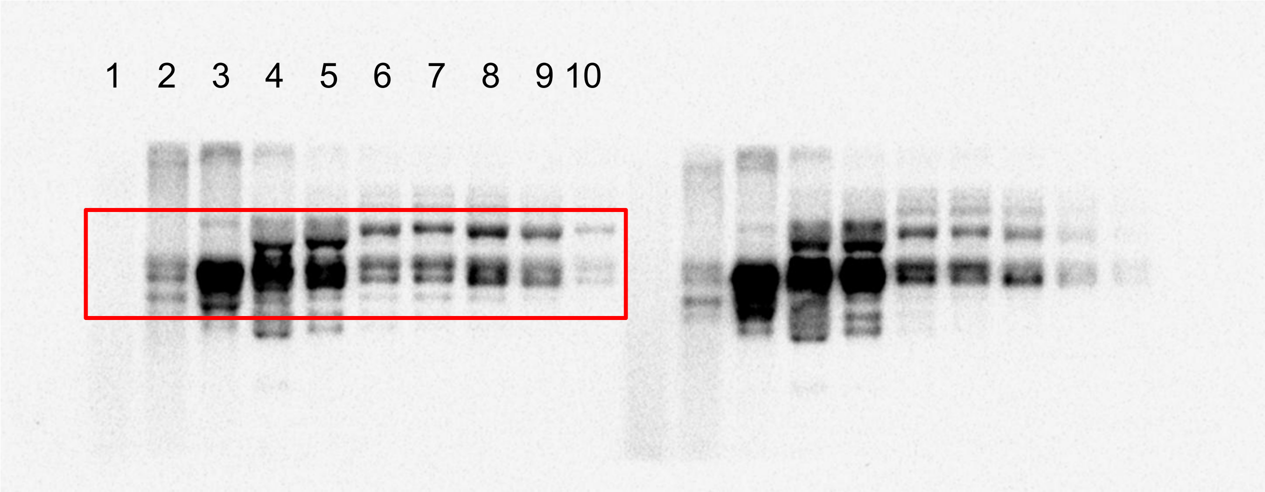

WT

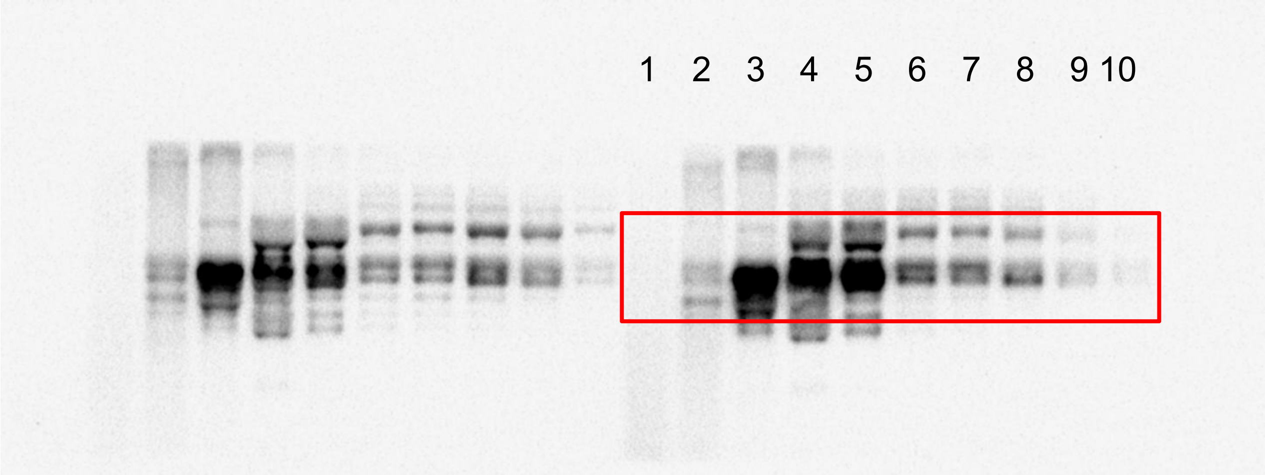

rbd1

psbC

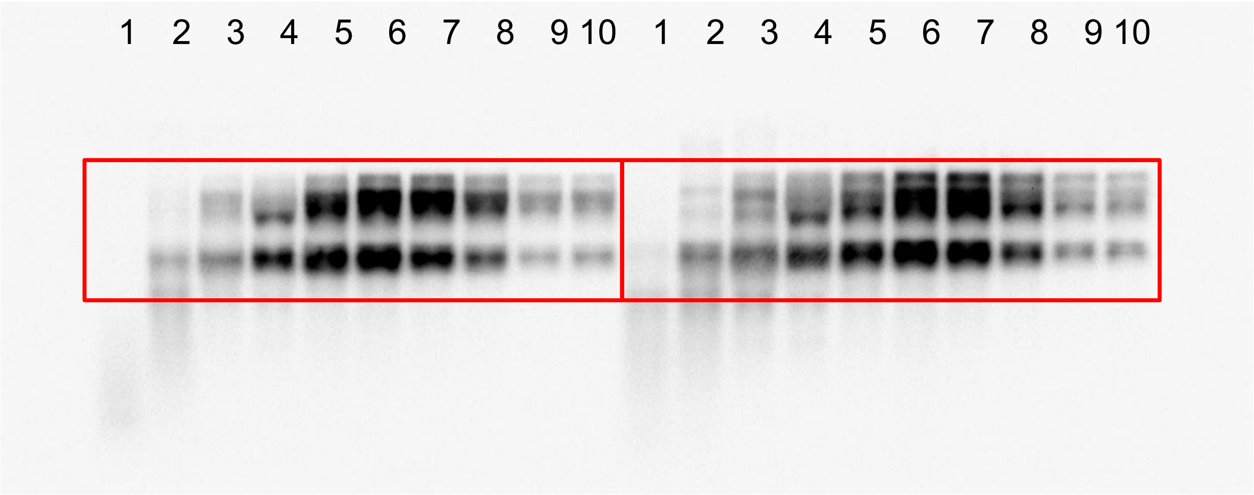

WT *rbd1*

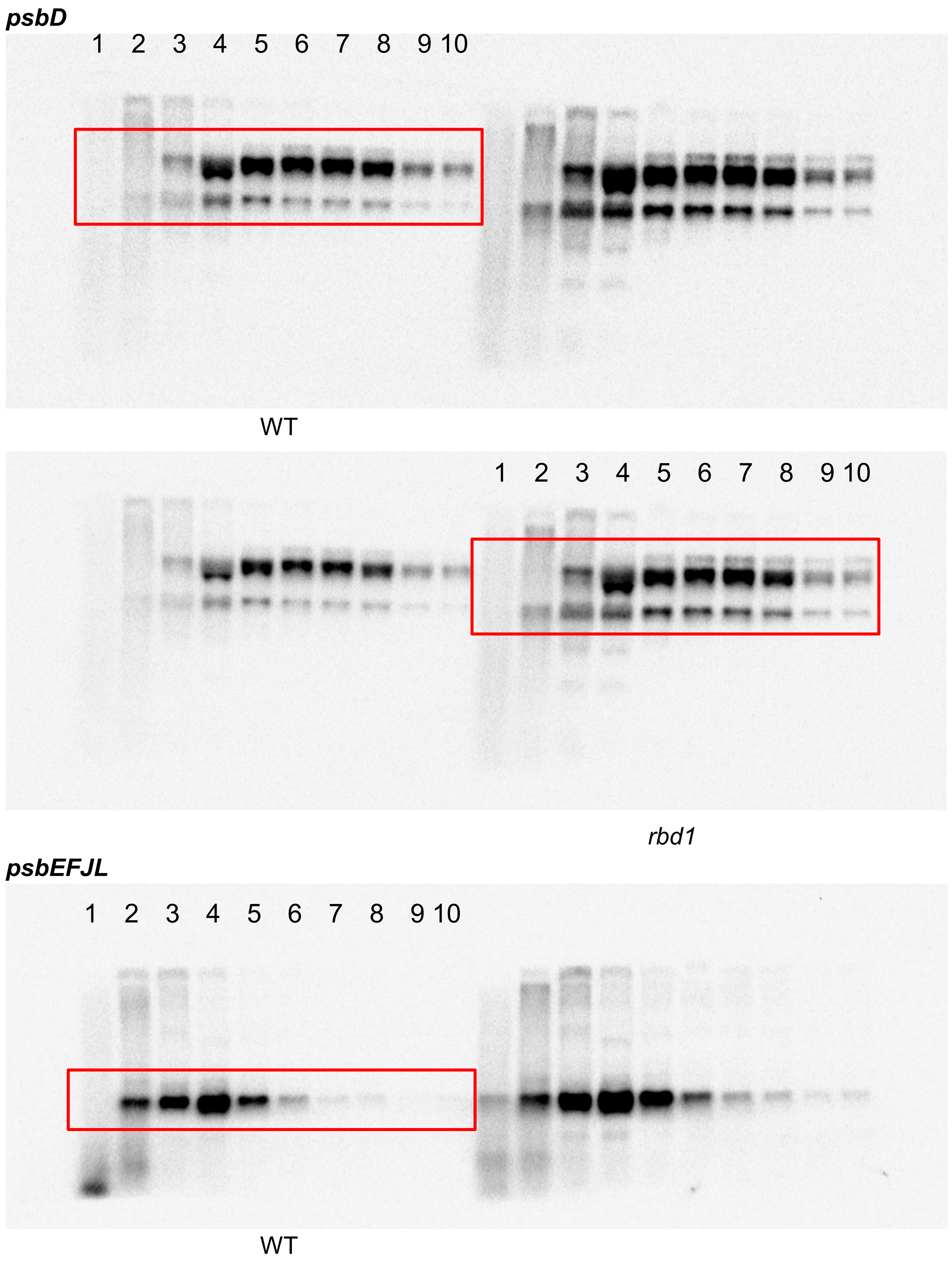
psbD
1 2 3 4 5 6 7 8 9 10
WT
1 2 3 4 5 6 7 8 9 10
rbd1
psbEFJL
1 2 3 4 5 6 7 8 9 10
WT

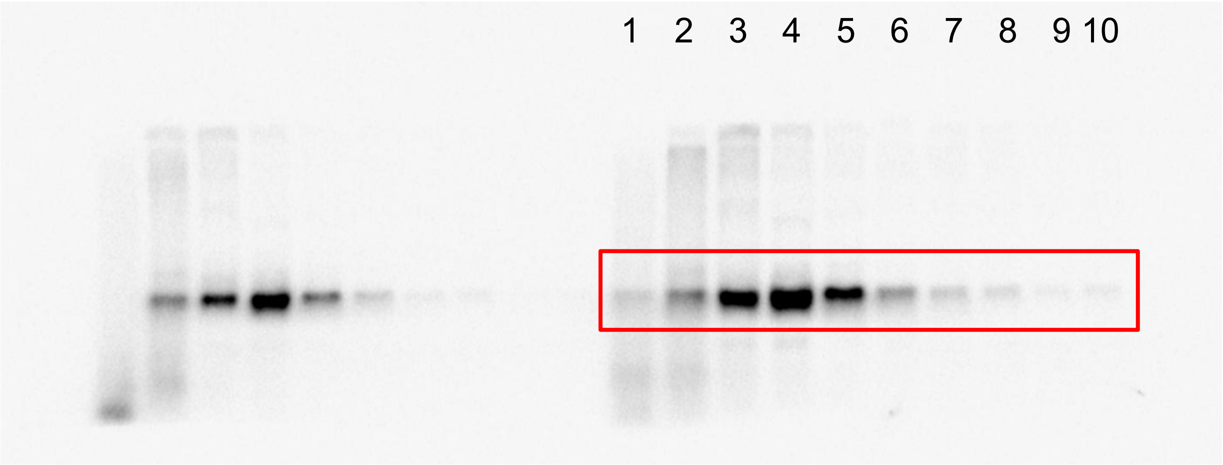

rbd1

psbKI

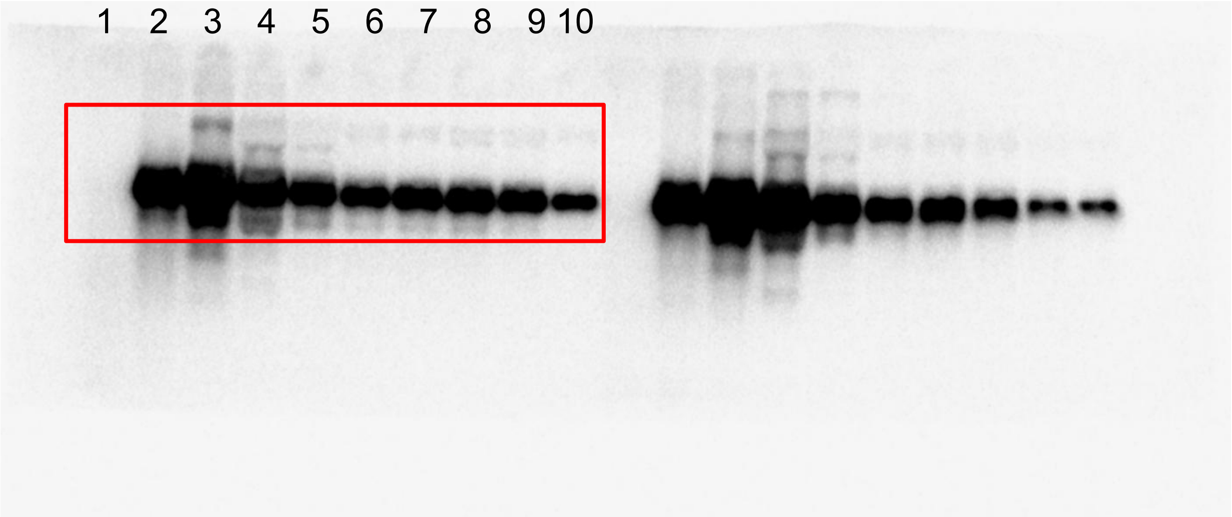

WT

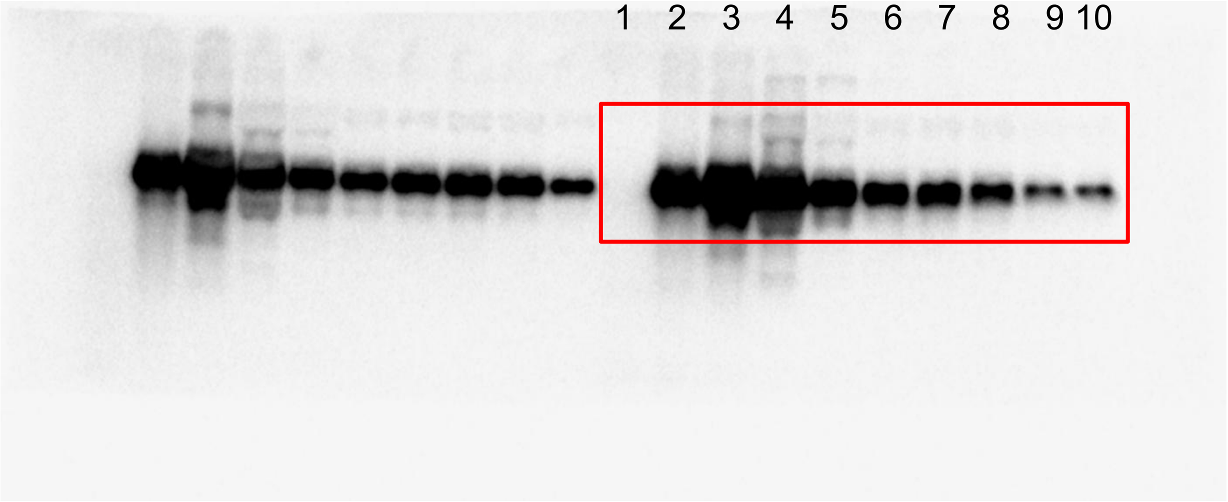

rbd1

Staining

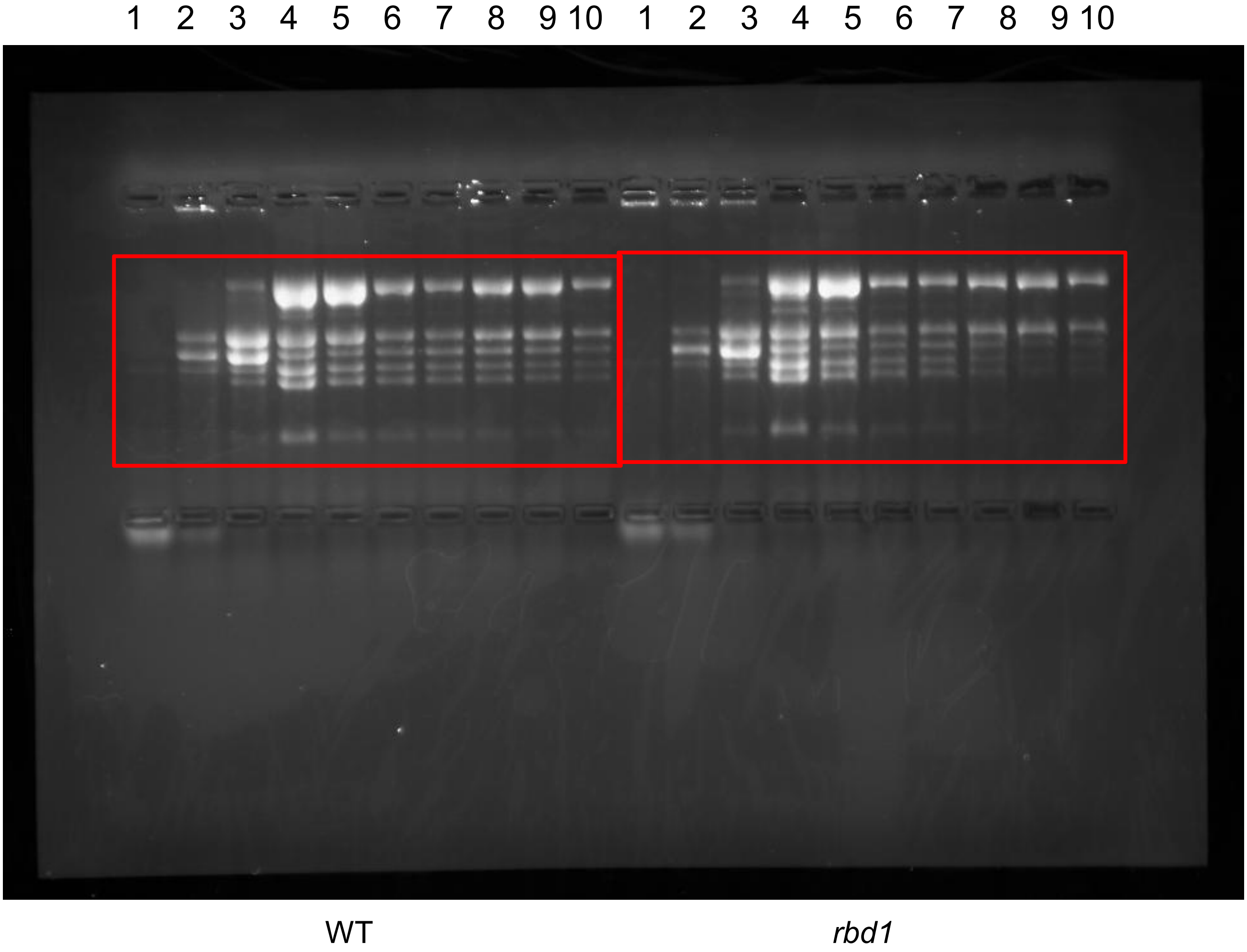

Figure 4C
psbA

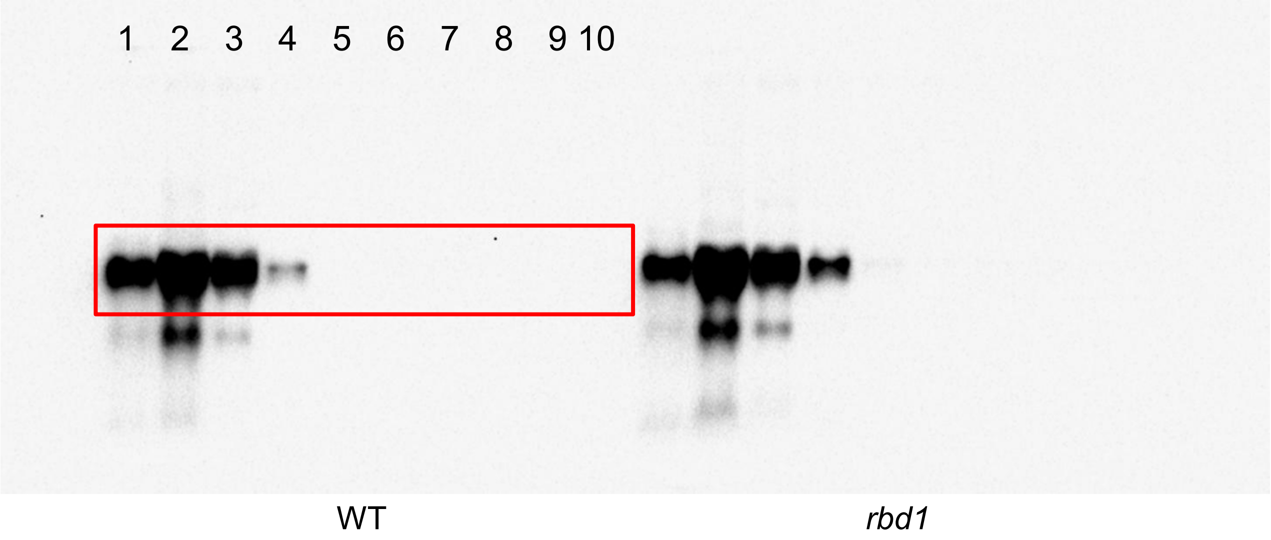

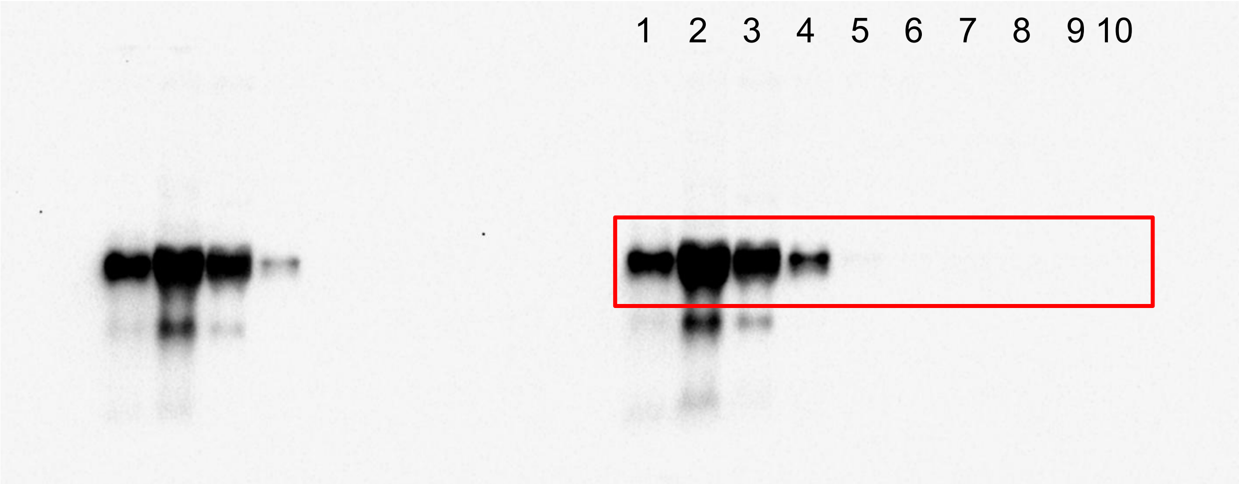

rbd1

Staining

1 2 3 4 5 6 7 8 9 10 1 2 3 4 5 6 7 8 9 10

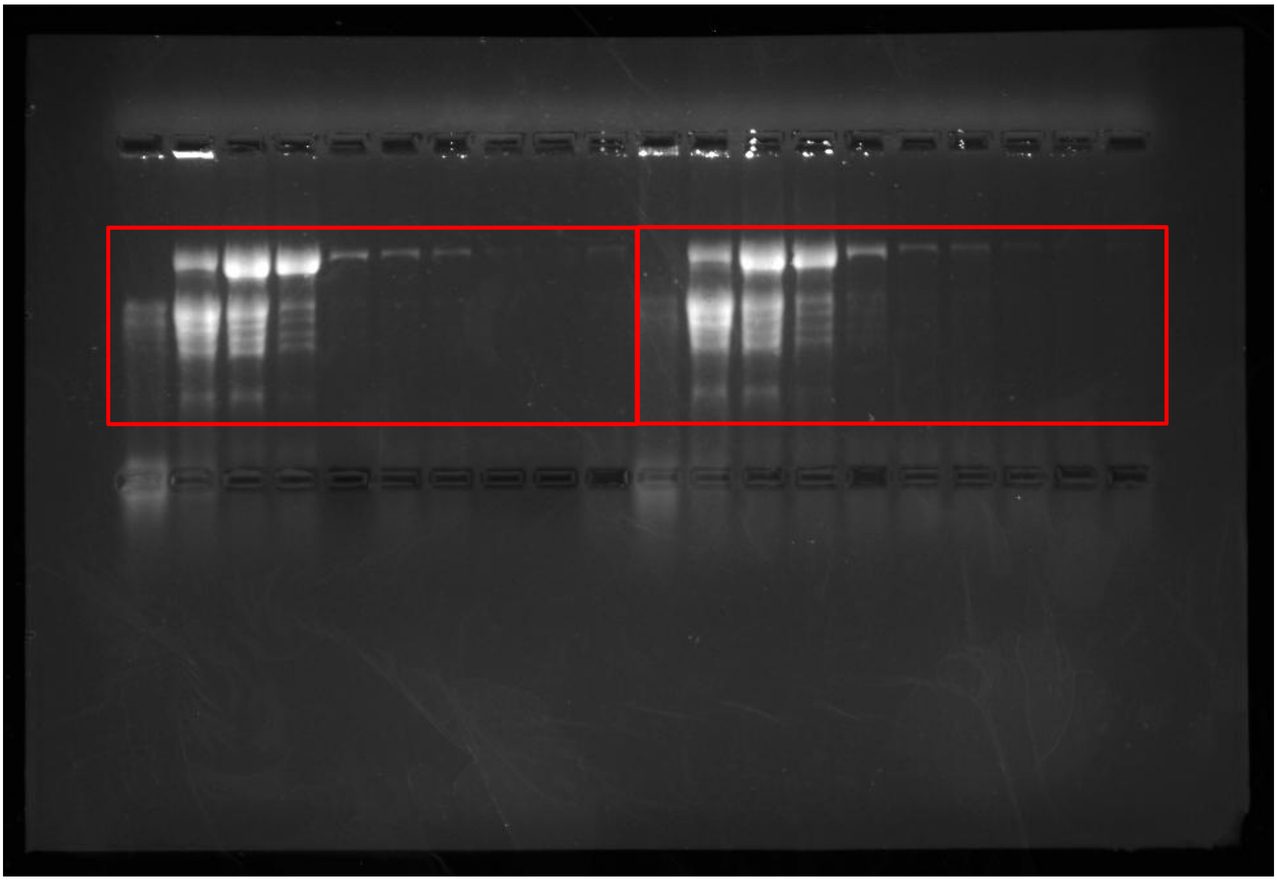

Supplement: Supplementary file 2 [file Data_Sheet_2.PDF]
